# Direct interaction of Plk4 with STIL ensures formation of a single procentriole per parental centriole

**DOI:** 10.1038/ncomms6267

**Published:** 2014-10-24

**Authors:** Midori Ohta, Tomoko Ashikawa, Yuka Nozaki, Hiroko Kozuka-Hata, Hidemasa Goto, Masaki Inagaki, Masaaki Oyama, Daiju Kitagawa

**Affiliations:** 1 Centrosome Biology Laboratory, Center for Frontier Research, National Institute of Genetics, Mishima, Shizuoka 411-8540, Japan; 2 Medical Proteomics Laboratory, Institute of Medical Science, The University of Tokyo, Minato-ku, Tokyo 108-8639, Japan; 3 Division of Biochemistry, Aichi Cancer Center Research Institute, Nagoya, Aichi 464-8681, Japan

## Abstract

Formation of one procentriole next to each pre-existing centriole is essential for centrosome duplication, robust bipolar spindle assembly and maintenance of genome integrity. However, the mechanisms maintaining strict control over centriole copy number are incompletely understood. Here we show that Plk4 and STIL, the key regulators of centriole formation, form a protein complex that provides a scaffold for recruiting HsSAS-6, a major component of the centriolar cartwheel, at the onset of procentriole formation. Furthermore, we demonstrate that phosphorylation of STIL by Plk4 facilitates the STIL/HsSAS-6 interaction and centriolar loading of HsSAS-6. We also provide evidence that negative feedback by centriolar STIL regulates bimodal centriolar distribution of Plk4 and seemingly restricts occurrence of procentriole formation to one site on each parental centriole. Overall, these findings suggest a mechanism whereby coordinated action of three critical factors ensures formation of a single procentriole per parental centriole.

## Abstract (web summary)

Centrosome duplication requires a cartwheel-shaped protein complex containing the protein HsSAS-6, which acts as a template for centriole assembly. Ohta *et al.* show that HsSAS-6 is recruited to this structure by STIL and Plk4, and reveal that Plk4 phosphorylates STIL, stabilizing its interaction with HsSAS-6.

## Graphical abstract

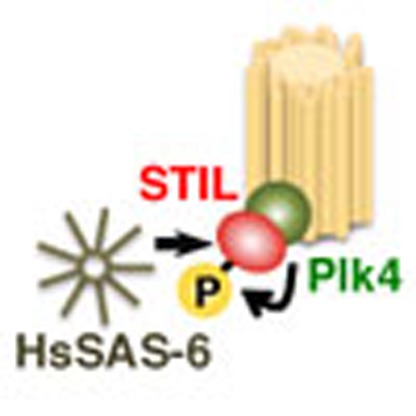

Centrosomes are the major microtubule organizing centre in most of animal cells and composed of a pair of centrioles surrounded by pericentriolar material. Centriole formation is indispensable for centrosome duplication and must be tightly coordinated with cell cycle progression to ensure robust formation of bipolar mitotic spindles and proper chromosome segregation. Centriole formation begins with the assembly of the cartwheel structure that mainly dictates the universal radial ninefold symmetry of centrioles, followed by attachment of peripheral centriolar microtubules and further centriole elongation^1^,^2^,^3^. Despite the recent notable progress in our understanding of the molecular and structural principles of centriole assembly, the mechanisms ensuring formation of only one procentriole at the base of each parental centriole per cell division cycle remain incompletely understood.

An evolutionarily conserved core pathway for centriole assembly includes the following five major components: Cep192/DSpd-2/SPD-2, Plk4/Sak/ZYG-1, HsSAS-6/DSas-6/SAS-6, STIL/Ana2/SAS-5 and CPAP/DSas-4/SAS-4 (refs 1, 2, 3). Among these components, Plk4 (refs 4, 5), HsSAS-6 (refs 6, 7) and STIL^8^,^9^,^10^,^11^ particularly may play an important role in controlling centriole number, since their overexpression induces concurrent formation of multiple procentrioles around a parental centriole^8^,^9^,^10^,^12^,^13^.

The conserved proteins of SAS-6 family are known to be a crucial element of a centriolar cartwheel structure^14^,^15^,^16^,^17^. Whereas it seems that nine of SAS-6 rod-shaped homodimers self-assemble into the central part of the cartwheel^14^,^15^,^16^, there could be additional factors strictly regulating this process or other cartwheel components facilitating SAS-6 self-assembly at the onset of procentriole formation. Given that Plk4 acts upstream of HsSAS-6 and STIL^8^,^9^,^10^,^12^,^13^, and also that HsSAS-6 and STIL appear to be interdependent for their loading to the centrioles^8^,^9^,^10^, Plk4 and STIL are plausible candidates for regulating HsSAS-6 oligomerization for cartwheel assembly. Although their relationship in other species appears to be mostly conserved^6^,^7^,^18^,^19^,^20^,^21^,^22^,^23^,^24^, how their collaborative action regulates the onset of centriole formation remains elusive. Moreover, the critical substrates of Plk4, a key kinase for centriole duplication, and how the kinase reaction triggers the onset of procentriole formation remain to be discovered.

In this study, we identify STIL as a critical substrate of Plk4, and show that the phosphorylation event leads to formation of the STIL/HsSAS-6 complex and initiation of procentriole assembly. Furthermore, we demonstrate negative feedback in which centriolar recruitment of the STIL/HsSAS-6 complex in turn limits distribution of centriolar Plk4 through the ubiquitin–proteasome pathway. This coordinated action of the three key factors triggers the onset of procentriole formation and, concurrently, restricts the occurrence of procentriole formation to one site per parental centriole.

## Results

### Plk4 recruits STIL to the centrioles by direct binding

**Figure 1:**
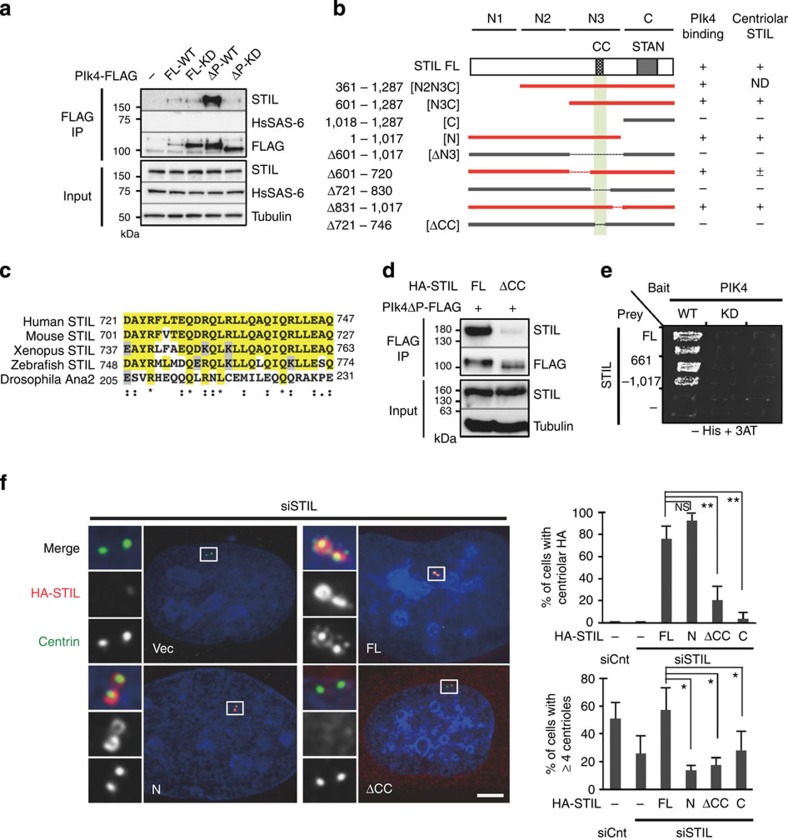
Direct interaction of Plk4 with STIL is required for centriolar targeting of STIL and centriole formation. (**a**) Co-immunoprecipitation (co-IP) assays testing interactions between the indicated Plk4-FLAG proteins and endogenous STIL. HEK293T cells expressing the empty FLAG vector (–), FLAG-tagged Plk4 full-length (FL) wild-type (FL-WT), FL kinase dead (FL-KD), ΔPEST (Δ272–311 a.a.) WT (ΔP-WT) or ΔPEST KD (ΔP-KD) were immunoprecipitated (IP) with FLAG antibodies. Total cell lysates and IPs were analysed by western blotting using STIL, HsSAS-6, FLAG or tubulin antibodies. (**b**) Schematic of HA-tagged STIL FL and deletion constructs used for co-IP assays with Plk4ΔPEST-FLAG in HEK293T cells. The right columns show a summary of the co-IP results and centriolar localization of the STIL constructs examined in U2OS cells. The STIL constructs that interact with Plk4ΔPEST-FLAG are represented in red and the minimal binding region in light green. The evolutionarily conserved coiled-coil (CC: a.a. 721–746) and STAN (a.a. 1,061–1,147) domains are indicated. ND, not determined. (**c**) Alignment of the CC domain within human, mouse, *Xenopus* and zebrafish STIL and *Drosophila* Ana2. Identical residues are coloured in yellow; similar residues in grey. Asterisks indicate the residues identical in all aligned sequences; colons: conserved substitutions; periods: semi-conserved substitutions. (**d**) HEK293T cells co-expressing Plk4ΔPEST-FLAG and HA-STIL FL or STILΔCC were IP with FLAG antibodies. Total cell lysates and IPs were analysed by western blotting using the indicated antibodies. (**e**) Yeast two-hybrid assay testing interactions between FL or the indicated fragment (a.a. 661–1,017) of STIL and WT or KD Plk4. The empty vectors (–) were used for negative controls. Two independent clones were grown on the plates without histidine and containing 50 mM 3-AT (Supplementary Fig. 1g). (**f**) U2OS cells were treated with control siRNA (siCnt) or siRNA targeting 3′UTR of endogenous STIL (siSTIL), followed by transfection with an empty vector (vec; –), HA-STIL FL, N-terminal fragment (N), ΔCC or C-terminal fragment (C). The cells were immunostained with antibodies against HA and centrin. DNA is shown in blue. Insets show approximately fivefold magnified views around the centrosome. Scale bar, 5 μm. Histograms represent frequency of interphase cells with centriolar HA (top) or with ≥4 centrin foci (bottom) in each condition. Values are mean percentages±s.d. from three independent experiments (*N*>50 for each condition). *P<0.05, **P<0.01, NS, not significant (one-tailed *t*-test).

First, to investigate the physical interactions between the three key factors for centriole assembly, we conducted co-immunoprecipitation analysis with human 293T cells expressing FLAG-tagged full-length Plk4 or Plk4ΔPEST lacking the first PEST destruction motif^25^, and tested whether the Plk4 proteins interact with endogenous STIL or HsSAS-6 proteins. This analysis revealed that Plk4-FLAG full-length interacts with endogenous STIL, but not with HsSAS-6 (Fig. 1a). Furthermore, we found that a higher amount of endogenous STIL could be co-immunoprecipitated with Plk4ΔPEST-FLAG owing to the increase in the expression levels of Plk4ΔPEST-FLAG compared with those of Plk4-FLAG full length (Fig. 1a). However, we could not detect such a robust interaction in the case of a kinase-dead mutant of Plk4ΔPEST-FLAG, suggesting that STIL preferentially interacts with Plk4 wild-type (WT) rather than the kinase dead. We also found that the interaction requires the two tandem polo boxes, PB1 and PB2, but not the C-terminal PB3 of Plk4 (Supplementary Fig. 1a–d)^5^,^26^. Using human influenza hemagglutinin (HA) tagged deletion constructs of STIL, we narrowed down the STIL domain that is required for Plk4 binding to the short conserved coiled-coil domain^23^ (Fig. 1b–d and Supplementary Fig. 1e,f). Moreover, yeast two-hybrid and *in vitro* binding assays established that Plk4 directly bound to the STIL fragments containing the CC domain presumably in a kinase-activity-dependent manner (Fig. 1e and Supplementary Fig. 1g,h).

We therefore reasoned that the physical interaction between the two proteins might be needed for centriolar recruitment of STIL. We depleted endogenous STIL using short interfering RNAs (siRNAs) against the 3′-untranslated region (3′UTR) and expressed STIL full-length or deletion constructs tagged with HA at comparable levels in human U2OS cells (Supplementary Fig. 1a). We found that whereas centriole numbers were reduced in the majority of interphase cells upon depletion of endogenous STIL, expression of exogenous full-length STIL could functionally rescue this phenotype (~26% and ~57% of cells with ≥4 centrioles, respectively; Fig. 1f) and frequently induced formation of multiple procentrioles next to each pre-existing centriole. In contrast, expression of the N-terminal fragment of STIL rescued centriole formation in only ~13% of cells (Fig. 1f) even though this fragment efficiently localized to the centrioles, indicating that the C-terminal region of STIL is crucial for centriole formation. Importantly, we determined that STIL proteins lacking the CC domain did not localize to the centrioles (~21% of cells with centriolar STILΔCC, compared with ~76% of cells with centriolar STIL FL; Fig. 1f) and failed to rescue centriole formation. Overall, these findings indicate that STIL is recruited to the centrioles by direct binding to Plk4 through its conserved CC domain, and that this interaction is necessary for centriole formation.

### STIL STAN motif is crucial for HsSAS-6 centriolar targeting

**Figure 2:**
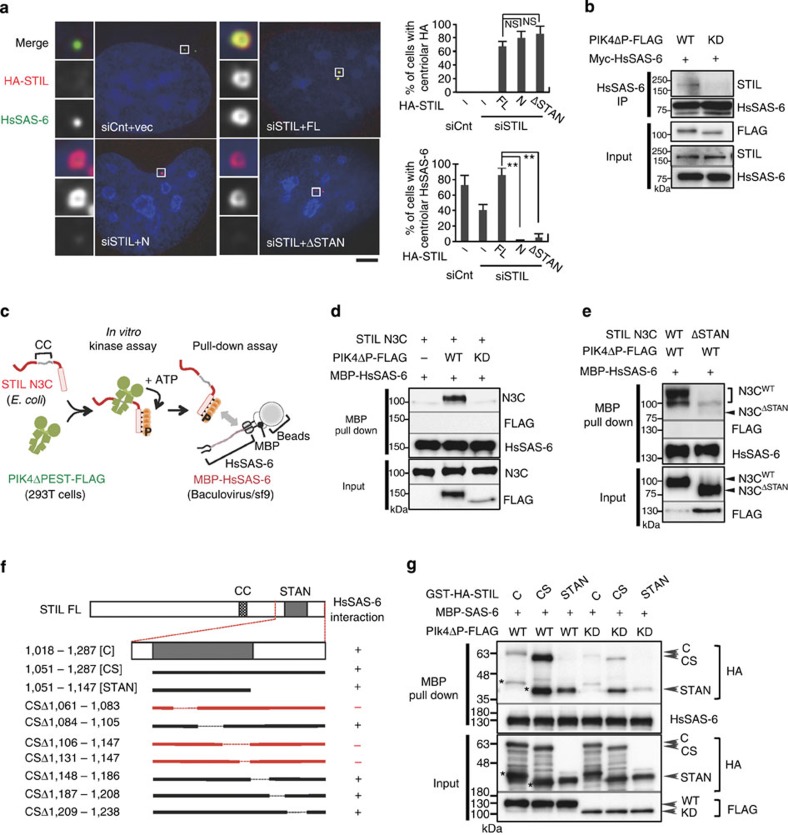
Plk4 kinase activity promotes the formation of a complex between STIL and HsSAS-6. (**a**) U2OS cells were treated with control siRNA or siRNA targeting 3′UTR of endogenous STIL, followed by transfection with an empty vector (–), HA-STIL full-length (FL), N (a.a. 1–1,017) or ΔSTAN (Δ1,061–1,147 a.a.). The cells were fixed and immunostained with antibodies against HA and HsSAS-6. DNA is shown in blue. Histograms represent frequency of interphase cells with centriolar HA (top) or with centriolar HsSAS-6 (bottom) in each condition. Insets show approximately sevenfold magnified views around the centrosome. Scale bar, 5 μm. Values are mean percentages±s.d. from three independent experiments (*N*>50 for each condition). *P<0.05, **P<0.01, NS, not significant (one-tailed *t*-test). (**b**) HEK293T cells co-expressing Plk4ΔP-FLAG wild type (WT) or kinase-dead (KD) and Myc-HsSAS-6 were immunoprecipitated (IP) with HsSAS-6. The amount of expressed Plk4ΔP-FLAG WT and KD was collected by IP using FLAG beads. Total cell lysates and IPs were analysed by western blotting using STIL, HsSAS-6 or FLAG antibodies. (**c**–**g**) *In vitro* reconstitution of a Plk4-dependent complex formation of STIL and HsSAS-6. (**c**) Schematic of the method used for monitoring the interaction between Plk4-phosphorylated STIL and HsSAS-6 *in vitro*. Plk4ΔP-FLAG WT or KD proteins were expressed and purified from HEK293T cell using anti-FLAG beads, followed by incubation with bacterially purified STIL N3C WT or ΔSTAN mutant (in **d**,**e**), deletion mutants of STIL C (in **f**,**g**) recombinant proteins for *in vitro* kinase assay. After the kinase reaction, the supernatant was collected and incubated with MBP-HsSAS-6 purified from baculovirus/insect cell expression system, and thereafter pulled down using amylose resin. Input and the protein complexes pulled down with amylose resin were analysed by western blotting using STIL, FLAG, HsSAS-6 and HA antibodies. Asterisks represent cleaved products of GST-HA-STIL C or CS.

We then investigated how the C-terminal region of STIL contributes to centriole formation. Since centriolar loading of HsSAS-6 is dependent on STIL^8^,^9^,^10^, we monitored whether HsSAS-6 proteins are present at centrioles when the C-terminal region or the conserved STAN motif^23^ of STIL was depleted. We first confirmed that whereas centriolar loading of HsSAS-6 was significantly reduced upon depletion of endogenous STIL, expression of STIL full length restored the localization of HsSAS-6 around the mother centriole (~40% and ~85% of cells with centriolar HsSAS-6, respectively; Fig. 2a). Intriguingly, we found that the STIL proteins lacking the STAN motif failed to recruit HsSAS-6 to the centrioles even though they robustly localized to the centrioles (~5% of cells with centriolar HsSAS-6; Fig. 2a and Supplementary Fig. 2a,b). These data indicate that the STAN motif is crucial for centriolar targeting of HsSAS-6.

### Plk4 kinase activity promotes STIL/HsSAS-6 interaction

It has been shown that the functional homologues of STIL: SAS-5 in *Caenorhabditis elegans*^7^ and Ana2 in *Drosophila melanogaster*^23^, can directly bind to SAS-6 proteins. However, in recent studies, a direct interaction between STIL and HsSAS-6 could not be detected^9^,^10^. We therefore hypothesized that Plk4 might regulate the mode of their interaction in human cells. To test this idea, we conducted co-immunoprecipitation experiments using 293T cells expressing Plk4ΔPEST-FLAG and Myc-HsSAS-6 proteins. Interestingly, we found that endogenous STIL was detected in the immunoprecipitated fraction of HsSAS-6 in cells expressing Plk4ΔPEST-FLAG and Myc-HsSAS-6, whereas this was not the case in cells expressing kinase-dead Plk4ΔPEST-FLAG and Myc-HsSAS-6 (Fig. 2b). This result prompted us to address whether phosphorylation of STIL by Plk4 facilitates a direct interaction between STIL and HsSAS-6.

To examine this, we used a combined biochemical approach using *in vitro* kinase assays with recombinant STIL N3C and Plk4ΔPEST-FLAG, followed by *in vitro* pull-down assays with recombinant maltose-binding protein (MBP) tagged HsSAS-6 (Fig. 2c). We found that Plk4ΔPEST efficiently phosphorylates STIL N3C *in vitro* (Supplementary Fig. 3c). Remarkably, we demonstrated that the phosphorylated STIL N3C directly bound to MBP-HsSAS-6 *in vitro* (Fig. 2d and Supplementary Fig. 2c,d). By contrast, this interaction was abolished when the STAN motif was removed from STIL N3C (Fig. 2e). This is in line with the observation that the STAN motif is crucial for centriolar loading of HsSAS-6 (Fig. 2a). To further narrow down the region of STIL for directly binding to HsSAS-6, we generated several deletion mutants within the C-terminal fragment of STIL (Fig. 2f,g and Supplementary Fig. 2e). As expected, amino acid (a.a.) 1,061–1,083 and a.a. 1,106–1,147 regions in the STAN appeared to be required for the interaction between the phosphorylated STIL and HsSAS-6 (Fig. 2f and Supplementary Figs 2e and 3a). Furthermore, we demonstrated that the STIL STAN motif phosphorylated by Plk4 is sufficient for binding to HsSAS-6 (Fig. 2g). Overall, these findings support the notion that phosphorylation of STIL by Plk4 facilitates the direct interaction between the conserved STAN motif of STIL and HsSAS-6, leading to centriolar loading of HsSAS-6.

### STIL is phosphorylated by Plk4 *in vitro* and *in vivo*

**Figure 3:**
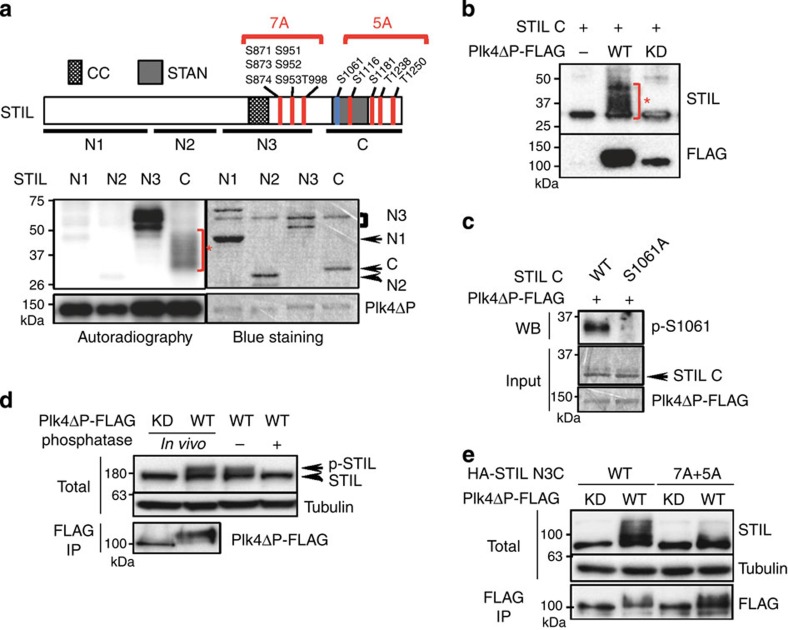
STIL is phosphorylated by Plk4 *in vitro* and *in vivo*. (**a**) Schematic of STIL full length and the summary of Plk4-phosphorylated serine/threonine residues of STIL identified by MS (top, indicated in red) and phospho-specific antibodies (top, indicated in blue; Supplementary Fig. 4a). STIL N3 and C fragments were phosphorylated by Plk4 *in vitro* (bottom). Recombinant STIL fragments N1 (a.a. 1–360), N2 (a.a. 361–600), N3 (a.a. 601–1,000) and C (a.a. 1,018–1,287) were bacterially purified and were incubated with Plk4ΔP-FLAG proteins purified from HEK293T cells for *in vitro* kinase assay. The incorporation of [γ-^32^P] ATP to the substrates was visualized by autoradiography, and the loaded proteins were monitored by Simply Blue Safestaining. CC (coiled-coil): a.a. 721–746; STAN: a.a. 1,061–1,147. Asterisk indicates a mobility shift of STIL C by hyper-phosphorylation. (**b**) *In vitro* kinase assay with STIL C fragment and Plk4ΔP-FLAG wild-type (WT) or kinase-dead (KD) as in **a**. After the kinase reaction, the resulting materials were analysed by western blotting using STIL or FLAG antibodies. Asterisk indicates a mobility shift of STIL C by hyper-phosphorylation. (**c**) Plk4 phosphorylates STIL at Serine 1061 *in vitro*. *In vitro* kinase assay was done as in **a** with the recombinant STIL C WT or S1061A proteins. The total reaction mixture was analysed by SDS–PAGE, followed by Simply Blue Safestaining or western blotting using antibodies against phospho-S1061. Note that the peptide containing S1061 was too large (53 a.a.) to be detected by MS. We therefore generated a phopho-specific antibody against S1061 to verify the existence of phosphorylation at this site. (**d**) Phosphatase assay of endogenous STIL in U2OS cells. Cells expressing Plk4ΔP-FLAG WT or KD were just lysed (first two lanes) or further treated with λ-phosphatase (±) (last two lanes). The resulting materials were analysed by western blot using antibodies against STIL or tubulin. The amount of expressed Plk4ΔP-FLAG WT and KD was collected by immunoprecipitate (IP) using FLAG beads, followed by western blot using FLAG antibodies. (**e**) U2OS cells expressing HA-STIL N3C WT or 7A+5A mutant and Plk4ΔPEST-FLAG WT or KD were lysed and analysed by western blot as in **d**.

We next sought to analyse the phosphorylation of STIL by Plk4 to further investigate its biological relevance for centriole formation. We first conducted *in vitro* kinase assays with Plk4ΔPEST-FLAG and the indicated four STIL fragments, and found that the fragments STIL N3 and C were efficiently phosphorylated by Plk4ΔPEST-FLAG *in vitro* (Fig. 3a). Intriguingly, we found a significant shift in the mobility of the phosphorylated STIL C fragment due to hyper-phosphorylation by Plk4ΔPEST (Fig. 3a,b). Using mass spectrometry (MS) analysis and phospho-specific antibodies, we identified seven and five phosphorylated serine/threonine residues within the N3 and C fragments, respectively (Fig. 3a,c and Supplementary Fig. 3a–c). We next investigated whether STIL is also phosphorylated by Plk4 *in vivo*. We observed a shift in the mobility of endogenous STIL when expressing Plk4ΔPEST-FLAG in U2OS cells (Fig. 3d). The shift was abolished upon treatment of the cell lysate with λ-phosphatase, indicating that endogenous STIL proteins were phosphorylated by Plk4 *in vivo* (Fig. 3d). Similarly, we found a significant band shift of STIL N3C fragment in U2OS cells expressing Plk4ΔPEST-FLAG, which is suggestive of the occurrence of multiple phosphorylations on the STIL fragment (Fig. 3e). Importantly, in addition, the shift of STIL N3C was drastically attenuated by mutating all the identified phosphorylated residues to alanine, indicating that these sites can be phosphorylated *in vivo* (Fig. 3e).

We then set out to examine the biological relevance of these phosphorylation sites for STIL function. We first found that the mutation of all seven S/T residues in the STIL N3 region or all five S/T residues in the STIL C region to alanine did not affect the interaction between Plk4 and STIL (Supplementary Fig. 4a–d,f). Moreover, since the N3 region of STIL is sufficient for binding to Plk4 (Fig. 1e), we further analysed deletion mutants within the N3 and confirmed that the conserved coiled-coil domain is essential for the Plk4/STIL interaction (Fig. 1b–d and Supplementary Fig. 4a–c,f). However, when mutating the sole S/T residue, T727, within the CC to alanine, we still detected the Plk4/STIL interaction (Supplementary Fig. 4a,g). These data suggest that while Plk4 kinase activity itself seems to be critical for the interaction, the phosphorylation of STIL by Plk4 is possibly dispensable for the interaction. We speculate that Plk4 autophosphorylation^27^,^28^ can modulate the state of Plk4 self-assembly, leading to the direct binding to the CC domain of STIL.

### Critical sites in the STAN for HsSAS-6 centriolar targeting

**Figure 4:**
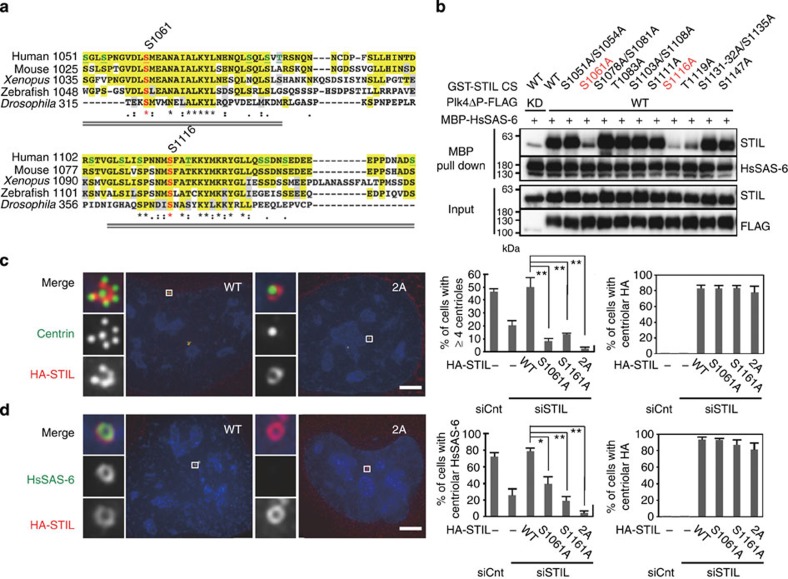
Critical sites in the STIL STAN motif for STIL/HsSAS-6 interaction and centriolar targeting of HsSAS-6. (**a**) Alignment of the STAN motif within human, mouse, *Xenopus* and zebrafish STIL and *Drosophila* Ana2. Colours and symbols indicate the same as in Fig. 1c. The S/T sites tested in **b** are shown in green. S1061 and S1116 are highlighted in red. Grey double lines indicate the critical parts of the STAN motif identified in Supplementary Fig. 2e. (**b**) Alanine mutational scan for the S/T sites within the STAN motif. *In vitro* kinase and binding assays were performed as described in Fig. 2c except for using recombinant GST-STIL CS wild-type (WT) or non-phosphorylatable alanine mutants. (**c**,**d**) U2OS cells were treated with control siRNA (siCnt) or siRNA targeting 3′UTR of endogenous STIL (siSTIL), followed by transfection with an empty vector (–), HA-STIL WT or non-phosphorylatable mutants, 2A (mutated at S1061 and S1116 to alanine), S1061A and S1116A. The cells were fixed and immunostained with antibodies against HA and centrin (**c**) or HsSAS-6 (**d**). DNA is shown in blue. Histograms represent frequency of interphase cells with ≥4 centrioles (**c**) or with centriolar HsSAS-6 (**d**) in each condition. Insets show approximately sevenfold magnified views around the centrosome. Scale bar, 5 μm. Values are mean percentages±s.d. from three independent experiments (*N*>50 for each condition). *P<0.05, **P<0.01; NS, not significant (one-tailed *t*-test).

Given that the phosphorylated STAN motif of STIL itself is sufficient for interacting with HsSAS-6 *in vitro* (Fig. 2f,g), we reasoned that S1061 and S1116 that are within the STAN motif and phosphorylated by Plk4 *in vitro* could be critical residues to mediate the STIL/HsSAS-6 interaction (Fig. 4a). To address this idea, we first conducted an alanine mutational scan for the S/T sites within the STAN motif, which led to the identification of three critical sites (S1061, S1116 and T1119) for the STIL/HsSAS-6 interaction *in vitro* (Fig. 4b and Supplementary Fig. 5a). As we could not find any evidence of phosphorylation at T1119 by MS, we assume that T1119 could be important for the structural arrangement of the STAN motif rather than being a phosphorylation site. Interestingly, S1061 and S1116 are the specific residues that are highly conserved from human STIL to *Drosophila* Ana2 (Fig. 4a), implying their biological significance throughout evolution. Second, we investigated whether alanine substitution mutants of the two phosphorylation sites of STIL have the ability to rescue the defect in the centriolar loading of HsSAS-6 and centriole formation when endogenous STIL proteins are depleted. Strikingly, we found that although mutating both residues to alanine did not affect centriolar targeting of STIL (Fig. 4c,d), expression of the S1061A, S1116A or 2A double mutant of STIL failed to rescue centriolar loading of HsSAS-6 and centriole formation in the cells depleted of endogenous STIL (centriolar HsSAS-6; ~4% for STIL 2A and ~78% for STIL WT: ≥4 centrin foci; ~2% for STIL 2A and ~50% for STIL WT; Fig. 4c,d and Supplementary Fig. 5b–f). On the other hand, expression of the STIL alanine mutants at three other MS-identified phosphorylation sites (S1181, T1238 or T1250) or the STIL deletion mutant lacking all the seven MS-identified phosphorylation sites in the N3 region mostly rescued the phenotype provoked by depletion of endogenous STIL (Supplementary Figs 4e and 5d–f). To further characterize the two critical phosphorylation sites in STIL, S1061 and S1116, we generated phosphomimetic mutants at these residues and investigated their function for centriole formation. Importantly, we found that expression of a phosphomimetic STIL mutant at S1061 under an attenuated human cytomegalovirus (CMV) promoter induced centriole overduplication more efficiently than that of STIL WT in the same condition (Supplementary Fig. 6a,b). Consistently, a phosphomimetic mutant at S1061 of the STIL STAN could interact with MBP-HsSAS-6 *in vitro* even without being phosphorylated by Plk4 (Supplementary Fig. 6c). On the other hand, introducing of a phosphomimetic mutation at S1116 impaired Plk4-mediated STIL/HsSAS-6 interaction *in vitro*, and centriolar targeting of HsSAS-6 in human cells (Supplementary Fig. 6d and data not shown). Given that the MS analysis revealed that this residue was phosphorylated by Plk4 *in vitro* and also that it is found to be phosphorylated in mammalian cells (PhosphoSitePlus database), we favour the possibility that substitution of S1116 to D/E could not mimic the phosphorylation state, but rather reduced the function of STIL. However, alternatively, it is also possible that S1116 is just critical for structural integrity of the STAN motif and/or its function independently of being phosphorylated. Taken together, we propose that S1061 and S1116 of STIL can be the most important phosphorylation sites for the STIL/HsSAS-6 interaction and resulting centriolar targeting of HsSAS-6 in procentriole formation.

### Bimodal centriolar distribution of Plk4 during cell cycle

**Figure 5:**
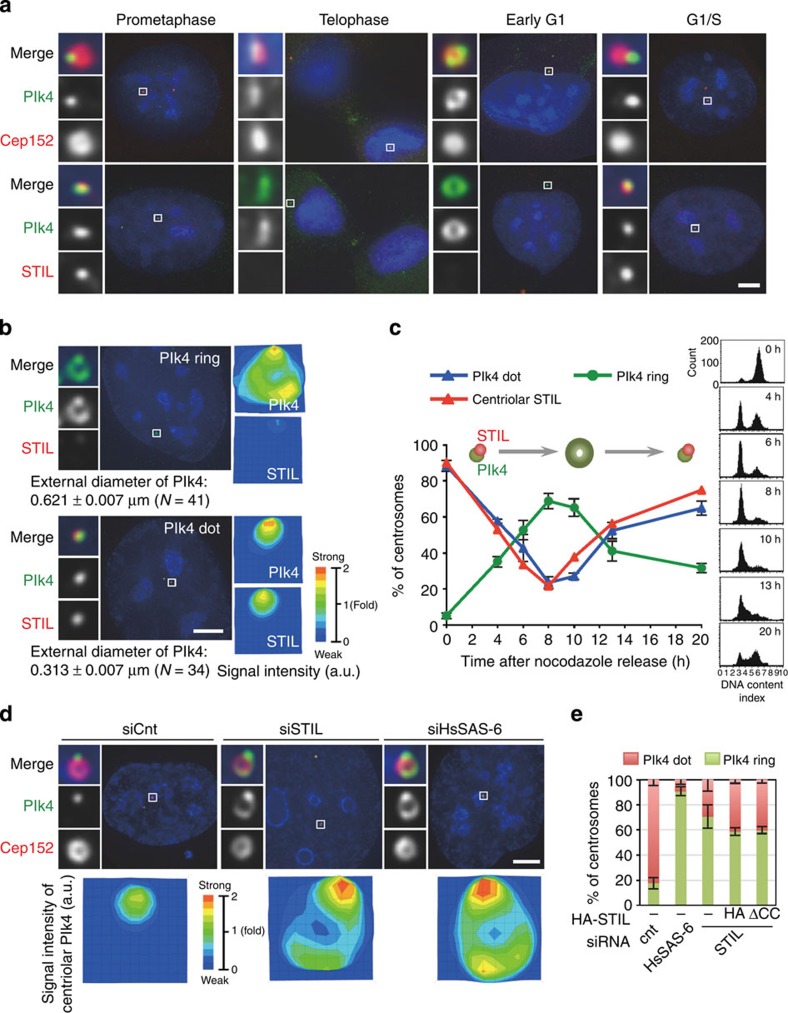
Bimodal centriolar distribution of Plk4 across the cell cycle. (**a**) Centriolar distribution of endogenous Plk4 proteins across the cell cycle. U2OS cells were arrested in prometaphase by treatment with nocodazole for 14 h. The cells were then washed out with fresh media without nocodazole three times and released from the arrest. After the release, the cells were fixed at different phases of the cell cycle and stained with antibodies against Cep152 or STIL as well as Plk4. Insets show ~10-fold magnified views around the centrosome. Scale bar, 5 μm. (**b**) Quantification of the local signal intensity of centriolar Plk4 and STIL. The local signal intensity was visualized in the indicated colours. The external diameter of centriolar Plk4 rings or dots was measured. Insets show approximately eight-fold magnified views around the centrosome. Scale bar, 5 μm. Values are mean percentages±s.e.m. *N*, number of total centrosomes. (**c**) Based on the centriolar distribution of Plk4, centrosomes were placed into two categories (ring-like or dot). The relative representation of each category as well as centriolar STIL over time is shown in the graph. The DNA content of cells for each time point was monitored by flow cytometry (right panels). Values are mean percentages±s.e.m. from four independent experiments for Plk4 (*N*>60 for each time point) and from two independent experiments for STIL (*N*>120 for each time point). The schematic represents the typical patterns of centriolar Plk4 and STIL across the cell cycle. (**d**,**e**) STIL and HsSAS-6 regulate centriolar distribution of Plk4. (d) U2OS cells were treated with control siRNAs (siCnt) or siRNAs targeting the 3′UTR of HsSAS-6 (siHsSAS-6) or STIL (siSTIL), and stained with antibodies against Plk4 and Cep152. The local signal intensity of centriolar Plk4 was quantified and visualized as indicated (bottom panels). DNA is shown in blue. Insets show approximately ninefold magnified views. Scale bar, 5 μm. (**e**) Histogram represents percentages of centrosomes categorized based on the centriolar distribution of Plk4. Values are mean percentages±s.e.m. from three independent experiments (*N*>50 for each condition). For the rescue experiment, U2OS cells treated with siRNAs targeting STIL-3′UTR and expressing HA empty vector (HA), HA-STILΔCC (Δ721–746 a.a.) were stained with antibodies against Plk4 and HA (Supplementary Fig. 7e).

We next hypothesized that the interaction between Plk4 and STIL might have an influence on the centriolar recruitment and/or maintenance of Plk4. To explore this possibility, we examined the distribution of endogenous Plk4 (ref. 29) in U2OS cells released from a nocodazole arrest and fixed at successive time points. Intriguingly, we found a bimodal distribution of Plk4 at centrioles depending on the cell cycle progression (Fig. 5a–c, Supplementary Figs 1b and 7a,b). As previously reported^30^,^31^, we observed that most of the cells exhibited one intense focus of Plk4 on each parental centriole during prophase/metaphase (~90%, ~0.3 μm in diameter; Fig. 5a–c and Supplementary Fig. 7a,b), and also that the signal intensity of the foci substantially declined around telophase. However, in the next cell cycle, we detected endogenous Plk4 localized in a ring-like manner around the parental centrioles (~0.62 μm in diameter; Fig. 5a–c and Supplementary Fig. 7a) and partially overlapped with a Cep152 ring, as a marker for parental centrioles (Fig. 5a)^32^,^33^. Quantitative analyses indicated that the majority of cells in G1 phase harboured a ring-like pattern of endogenous Plk4 (~65% at 8–10 h after the release; Fig. 5a,c). Remarkably, we further found that the ring-like pattern of centriolar Plk4 changed back into a dot on the parental centriole wall in G1/S phase (~52% as a dot at 13 h after the release; Fig. 5a,c). Co-staining of endogenous Plk4 and STIL revealed that when STIL localized to centrioles in G1/S phase, centriolar distribution of Plk4 was always restricted to a spot and largely overlapped with centriolar STIL foci (Fig. 5a–c). We could hardly detect the existence of a ring-like pattern of Plk4 with STIL foci at centrioles, suggesting that they are mutually exclusive. In addition, we noted that STIL and HsSAS-6 appeared to be loaded to the centrioles almost concomitantly and precisely co-localized with each other (data not shown)^31^. We therefore assumed that the existence of STIL and HsSAS-6 might allow the conversion of the centriolar Plk4 ring into a dot.

To test this, we examined the centriolar distribution of Plk4 when STIL or HsSAS-6 was depleted from U2OS cells. Interestingly, we found that most of the interphase cells depleted of STIL or HsSAS-6 harboured the ring-like arrangement of centriolar Plk4 while the cell cycle progression was not affected (Plk4 ring; ~18% for siCnt, ~91% for siHsSAS-6 and ~71% for siSTIL; Fig. 5d,e, and Supplementary Figs 1a and 7c,d). In addition, the expression of STIL ΔCC in STIL siRNA-treated cells did not suppress the increase in the number of cells with a ring-like pattern of centriolar Plk4 (~59% for siSTIL+empty vector and ~60% for siSTIL+ΔCC; Fig. 5e and Supplementary Fig. 7e), indicating that the existence of STIL and HsSAS-6 at centrioles is required for the conversion of the centriolar Plk4 ring into a dot.

### The interaction of Plk4 with STIL protects centriolar Plk4

**Figure 6:**
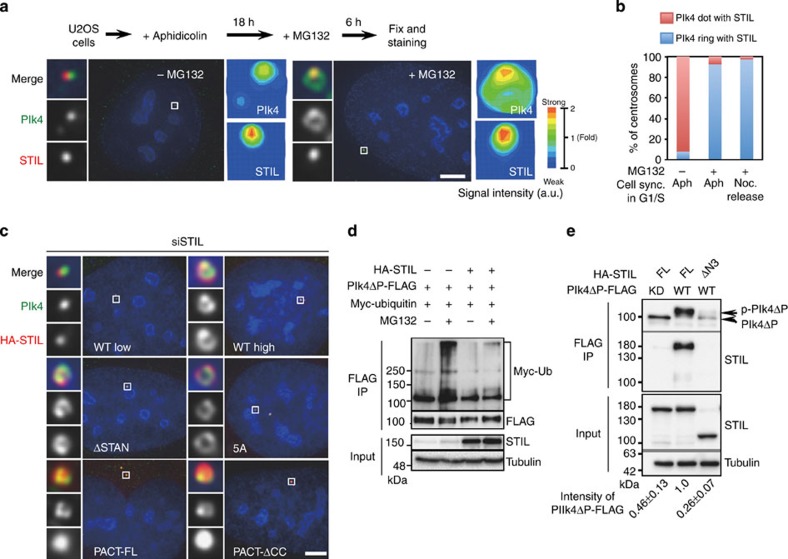
The interaction of Plk4 with STIL regulates the bimodal distribution of Plk4 around parental centriole. (**a**,**b**) U2OS cells were arrested in G1/S phase by treatment with aphidicolin (Aph) for 18 h, followed by addition of 10 μM MG132 (±) for 6 h. Cells were then fixed and stained with antibodies against Plk4 and STIL. DNA is shown in blue. In this figure, all insets show approximately ninefold magnified views around the centrosome. Scale bars, 5 μm. The local signal intensity of centriolar Plk4 and STIL was quantified and visualized as indicated. The percentages of centrosomes with Plk4 rings or Plk4 dots were quantified in **b** (*N*≥40 centrosomes with STIL from four independent experiment). Similar experiments were also performed with U2OS cells in G1/S phase using cell cycle synchronization (sync.) with nocodazole (Noc) treatment in **b**. (**c**) U2OS cells treated with siRNAs targeting STIL-3′UTR (siSTIL) and expressing HA-STIL full length at low or high levels, ΔSTAN (Δ1,061–1,147 a.a.), 5A non-phosphorylatable mutant, PACT-full length or PACT-ΔCC were stained with antibodies against Plk4 and HA. Note that the signal intensity of centriolar Plk4 ring in the absence of endogenous STIL or HsSAS-6 is relatively higher than that in the case of overexpression of STIL full length or mutants used in this experiment. The experiment was repeated at least three times. (**d**) Ubiquitination assay. HEK293T cells transfected with the indicated combination of plasmids were treated with 10 μM MG132 (±) for 6 h. The cells were then immunoprecipitated (IP) with FLAG antibodies. Total cell lysate and IPs were analysed by western blotting using Myc, FLAG, STIL or tubulin antibodies. (**e**) HEK293T cells co-expressing Plk4ΔPEST-FLAG wild-type or the kinase dead (KD) and HA-STIL full length or STILΔN3 were IP with FLAG antibodies. Total cell lysate and IPs were analysed by western blotting using STIL, FLAG or tubulin antibodies. The values on the bottom indicate the relative amount of IP Plk4ΔPEST-FLAG. Values are mean percentages±s.e.m. from three independent experiments.

Given that the expression levels of Plk4 are known to be regulated by trans-autophosphorylation that induces proteolytic degradation by the E3 ubiquitin ligase Skp-Cullin-F-box containing complex, SCF^Slimb^^/β-TrCP^ and the ubiquitin–proteasome-dependent pathway in *D. melanogaster* and mammalian cells^27^,^28^,^34^,^35^, we hypothesized that the conversion of centriolar Plk4 followed by centriolar STIL/HsSAS-6 loading might involve protein degradation through the ubiquitin–proteasome pathway mediated by the SCF complex or other E3 ligases. To address this, we synchronized U2OS cells in G1/S phase with aphidicolin and transiently treated with MG132, a proteasome inhibitor. In the cells treated without MG132, endogenous Plk4 localized mostly as a dot and resided with STIL foci at centrioles (Plk4 ring; ~8%; Fig. 6a,b and Supplementary Fig. 7f). In stark contrast, when treated with MG132, majority of the cells harboured a complete ring of Plk4 with STIL foci at the centrioles, which we could hardly detect in normal cycling cells (Plk4 ring; ~93%; Fig. 6a,b and Supplementary Fig. 7c,f). In this situation, Plk4 proteins were primarily enriched at the position overlapping with STIL foci as compared with the rest of the ring. This implies that centriolar STIL could interact with and protect Plk4 proteins from protein degradation, whereas the residual centriolar Plk4 proteins are normally degraded.

To address this model, we next investigated whether overexpression of STIL full-length or mutant proteins stabilizes centriolar Plk4. We found that whereas low expression of HA-STIL full length in the cells depleted of endogenous STIL restored the population of cells containing Plk4 dots at the centrioles, overexpression of HA-STIL full-length, ΔSTAN or 5A mutant proteins stabilized centriolar Plk4 as a ring overlapping with the STIL proteins (Fig. 6c). Considering that the centriolar loading of HsSAS-6 and procentriole formation were inhibited in the cells expressing HA-STIL ΔSTAN or 5A mutant, this result suggests that centriolar presence of STIL might be sufficient for stabilizing centriolar Plk4 in this situation. Intriguingly, when expressing the PACT-STIL ΔCC mutant that fails to interact with Plk4, but still localizes to centrioles, we found that expression of the STIL mutant proteins maintained centriolar Plk4 as a ring overlapping with the STIL mutant proteins and recruited HsSAS-6 to centrioles (Fig. 6c and Supplementary Fig. 7g). This result suggests that PACT-STIL ΔCC can bypass the requirement of STIL function for centriolar loading of HsSAS-6, and also that close-range presence of STIL and Plk4 at centrioles, or their transient interaction could be critical for protecting centriolar Plk4 from protein degradation and for centriolar recruitment of HsSAS-6. However, we cannot exclude the possibility that residual binding activity of overexpressed STIL ΔCC to Plk4 somehow managed to facilitate those events.

Using biochemical analysis in human 293T cells, we next sought to investigate whether STIL protects Plk4 from protein degradation. Importantly, we demonstrated that expression of STIL efficiently inhibited ubiquitination of Plk4ΔPEST-FLAG WT (Fig. 6d). Given that the first PEST domain of Plk4 contains the site recognized by the SCF complex^25^,^34^, the degradation of Plk4ΔPEST proteins might be regulated by another E3 ubiquitin ligase. Furthermore, we found that expression of STIL full-length stabilized Plk4ΔPEST-FLAG WT, whereas that was not the case when co-expressing STILΔN3, which lacks the binding region to Plk4 (Fig. 6e). We also noted that STIL seemed to stabilize activated Plk4ΔPEST-FLAG WT proteins (p-Plk4ΔP) (Fig. 6e and Supplementary Fig. 7h). This is in agreement with the observation that STIL preferentially interacts with Plk4 WT rather than the kinase dead (Fig. 1). Taken together, these data suggest that the interaction between STIL and Plk4 protects Plk4 from protein degradation mediated by the ubiquitin–proteasome pathway.

## Discussion

**Figure 7:**
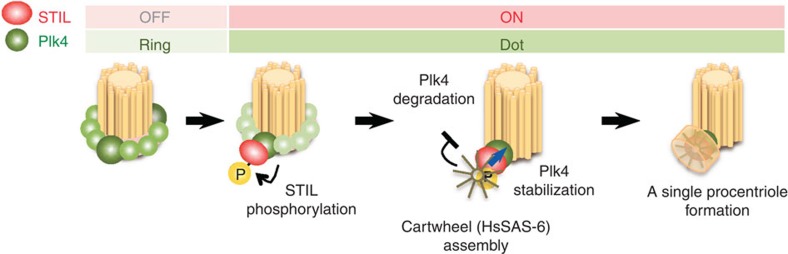
Model suggesting how the formation of a single procentriole per each parental centriole is controlled. Direct association of STIL with Plk4 and STIL phosphorylation by Plk4 occur at the onset of centriole formation. Phosphorylated STIL directly binds to HsSAS-6, which leads to centriolar loading of HsSAS-6 for cartwheel assembly. Centriolar loading of the STIL/HsSAS-6 complex limits centriolar distribution of Plk4 to one site per each parental centriole. This coordinated action could ensure formation of a single procentriole per each parental centriole and, concurrently, inhibit formation of another procentriole.

In conclusion, our findings uncover a molecular basis for the onset of centriole formation by demonstrating that direct association of STIL with Plk4 and STIL phosphorylation by Plk4 lead to centriolar loading of HsSAS-6 for cartwheel assembly (Fig. 7). Furthermore, our study suggests a negative feedback model in which centriolar STIL/HsSAS-6 recruitment limits centriolar distribution of Plk4 to one site per parental centriole (Fig. 7 and Supplementary Fig. 7i). This coordinated action promotes formation of a single procentriole and, concurrently, could inhibit formation of another procentriole, thus presumably contributing to maintenance of one procentriole next to each parental centriole.

How could phosphorylated STIL facilitate cartwheel assembly by direct binding to HsSAS-6? It could be structurally critical for the spatial arrangement and connection between HsSAS-6 homodimers. Indeed, it has been shown in *D. melanogaster* that co-overexpression of DSas-6 and Ana2 induces formation of highly ordered tubules with the structures reminiscent of the centriolar cartwheel hub^20^. However, to drive centriole overduplication through efficient centriolar recruitment of extra DSas-6 and Ana2, additional co-expression with Plk4/Sak is needed^20^. These observations are compatible with our findings, implying that the interplay between the three key factors that we demonstrated may underlie the centriole assembly pathway across species. On the other hand, in *C. elegans*, given that the interaction between SAS-5 and SAS-6 seems to be detectable presumably even in the absence of ZYG-1 (refs 7, 36), and also that ZYG-1 can directly bind to^37^ and phosphorylate SAS-6 (ref. 38) for centriole assembly, the regulatory mechanism for cartwheel assembly may be somehow different in this organism along with its structural divergence of the centriole structure.

It has been recently shown that the expression levels of Plk4 are regulated by trans-autophosphorylation that mediates proteolytic degradation by the E3 ubiquitin ligase SCF^Slimb/β-TrCP^ and the ubiquitin–proteasome-dependent pathway in *D. melanogaster*^39^,^40^ and mammalian cells^27^,^28^. The reduction of centriolar Plk4 followed by centriolar recruitment of the STIL/HsSAS-6 complex seems to involve protein degradation through the ubiquitin–proteasome pathway (Fig. 5d). It will be therefore important in the future study to further investigate the detailed mechanisms how the recruitment of STIL/HsSAS-6 allows SCF^Slimb/β-TrCP^ and/or other E3 ubiquitin ligases to target for degradation of centriolar Plk4 proteins that do not form a complex with STIL. Considering that physical association and co-localization of Plk4 and STIL at one site on the parental centriole wall is dependent on the kinase activity of Plk4, it is possible that a complex formation between active Plk4 and STIL prevents Plk4 from undergoing protein degradation. Alternatively, any other protein that STIL brings to centrioles may protect Plk4 from the protein degradation. Furthermore, given that the presence of the SAS-5/SAS-6 complex at centrioles is needed for the diminution of centriolar ZYG-1 during interphase^18^, it is tempting to speculate that the feedback mechanism that we demonstrated in this study is a conserved system for tight control of centriole copy number throughout evolution.

Based on the findings in this study, further study will be needed to establish the structural model how the sequential physical interactions between Plk4/STIL/HsSAS-6 proteins lead to the assembly of a core structure for initiating cartwheel assembly.

## Methods

### Cell culture and cell lines

Human U2OS and HEK293T cells were obtained from the European Collection of Cell Culture (ECACC). U2OS cells stably expressing GFP-centrin1 were gifted from Bornens^41^. These cells were cultured in DMEM supplemented with 10% fetal bovine serum at 37 °C in 5% CO_2_ incubator.

### Cell cycle synchronization and flow cytometry analysis

For cell synchronization at prometaphase, cells were treated with 100 ng ml^−1^ nocodazole for 14 h, washed three times with PBS and released in fresh medium. For cell cycle arrest in G1/S phase, cells were treated with 2 μg ml^−1^ aphidicolin for 24 h.

For flow cytometry analyses, cells cultured on dishes were trypsinized, washed twice with PBS and fixed in 70% cold ethanol at −20 °C at each time point. The fixed cells were washed with PBS twice and incubated with Muse Cell Cycle reagents at room temperature (RT) for 30 min. The DNA contents of the cells were then measured using Muse Cell Analyzer (Merck Millipore). Flow cytometry analysis was repeated at least two times.

### Molecular biology and RNA interference

The following siRNAs were used: Stealth siRNA (Life Technologies) against 3′UTR of HsSAS-6 (5′-GAGCUGUUAAAGACUGGAUACUUUA-3′) and negative control Low GC Duplex no. 2 (12935110); custom siRNA (Sigma Genosys) against 3′UTR of Plk4 (5′-CTCCTTTCAGACATATAAG-3′); custom siRNA (JBios) against 3′UTR of STIL (5′-GTTTAAGGGAAAAGTTATT-3′).

pcDNA3 constructs encoding Plk4 full-length FLAG, a kinase-dead Plk4 [K41M]-FLAG, Plk4Plk4ΔPEST-FLAG and Plk4[K41M]ΔPEST-FLAG were gifts from Dr Hiroyuki Mano. The mammalian expression constructs for HA-STIL full-length, deletion mutants, alanine substitution mutants and phosphomimetic mutants were created by insertion of subcloned fragments into SpeI-digested modified pCMV5-HA vector or using PrimeSTAR mutagenesis basal kit (TaKaRa). pcDNA3-Plk4ΔPEST–[ΔPB1]/[ΔPB2]/[ΔPB3]–FLAG expression constructs were created using PrimeSTAR mutagenesis basal kit (TaKaRa). Since expression levels of Plk4ΔPΔPB3-FLAG were high compared with those of the other Plk4 mutants used in Supplementary Fig. 1d, the Plk4ΔPΔPB3-FLAG plasmid was transfected into cells with half the amount of the other Plk4 plasmids.

Transfection of siRNA or DNA constructs into U2OS and HEK293T cells was performed using Lipofectamine RNAiMAX (Life Technologies) or Lipofectamine 2000 (Life Technologies) according to the manufacturer’s instructions. Unless otherwise noted, transfected cells were analysed 48–72 h after transfection with siRNA and 24 h after transfection with DNA constructs.

### Antibodies

The following primary antibodies were used in this study: rabbit polyclonal antibodies against STIL (Abcam, ab89314, indirect immnunofluorescence (IF) 1:500, western blotting (WB) 1:1,000), Cep152 (Bethyl Laboratories, A302-480A, IF 1:1,000), HA-tag (Abcam, ab9110, IF 1:1,000, WB 1:1,000); mouse monoclonal antibodies against centrin-2 (Millipore, 20H5, IF 1:1,000), HsSAS-6 (Santa Cruz Bio-technology, Inc., sc-81431, WB 1:1,000), Plk4 (Merck Millipore, clone 6H5, MABC544, IF 1:500), FLAG-tag (Sigma, F1804, IF 1:1,000, WB 1:1,000), HA-tag (Covance, MMS-101P, WB 1:500) and α-tubulin (Sigma, DM1A, WB 1:2,000). P-S1061 rabbit antibodies were raised against C+NGVDL[pS]MEAN, where [pS] is a phosphorylated serine residue (Eurofins Operon). The following secondary antibodies were used: Alexa Fluor 488 goat anti-mouse IgG (H+L) (Molecular Probes, 1:500), Alexa Fluor 568 goat anti-rabbit IgG (H+L) (Molecular Probes, 1:500) for IF; goat polyclonal antibodies horseradish peroxidase against mouse IgG (Promega, W402B, 1:5,000), rabbit IgG (Promega, W401B, 1:5,000) for WB.

### Indirect immunofluorescence and immunoblotting

For indirect immunofluorescence microscopy, the cells cultured on coverslips were fixed using −20 °C methanol for 10 min. The cells were then permeabilized with PBS/0.05% Triton X-100 (PBSX) for 5 min, washed with PBS three times and incubated for blocking in 1% BSA in PBSX for 30 min at RT. The cells were then incubated with primary antibodies for 3 h at RT, washed with PBSX three times and incubated with secondary antibodies for 1 h at RT. The cells were thereafter washed with PBSX twice, stained with 0.2 μg ml^−1^ Hoechst 33258 (Dojindo) in PBS for 5 min at RT, washed again with PBSX and mounted onto glass slides. Counting the number of immunofluorescence signals was performed by using an Axioplan2 fluorescence microscope (Carl Zeiss) with a × 100/1.4 numerical aperture plan-APOCHROMAT objective. Data acquisition for the images and quantification of the signal intensity were performed using DeltaVision Personal DV-SoftWoRx system (Applied Precision) equipped with a CoolSNAP CH350 CCD camera. The images were acquired as serial sections along the *z* axis and stacked using the ‘quick projection’ algorithm in SoftWoRx. The signal intensities of centriolar Plk4, STIL and HsSAS-6 were quantified using the Data Inspector tool in SoftWorx. The captured images were processed with Adobe Photoshop CS5.1 (version 12.1). We assessed cells from several fields for each experiment, and we were normally blinded to the sample ID during experiments and outcome assessment. Once a field was determined, we counted all cells that matched with the criteria within the field. In the experiments using the cells expressing HA-tagged full-length or mutants of STIL, we counted cells adequately expressing the STIL proteins at comparable levels and excluded cells expressing the STIL proteins at low levels or cells excessively expressing the STIL proteins.

For preparation of human cell lysates for immunoblotting, cells were collected, washed in PBS and lysed by vortexing at 4 °C in lysis buffer (20 mM Tris/HCl pH 7.5, 50 mM NaCl, 1% Triton X-100, 5 mM EGTA, 1 mM dithiothreitol (DTT), 2 mM MgCl_2_ and 1/1,000 protease inhibitor cocktail (Nakalai Tesque)). Lysates were cleared by centrifugation for 10 min at 13,000 r.p.m. at 4 °C and the supernatant was collected. SDS–polyacrylamide gel electrophoresis (SDS–PAGE) was performed using 7–12% polyacrylamide gels, followed by transfer on Immobilon-P membrane (Millipore Corporation). The membrane was probed with the primary antibodies, followed by incubation with their respective horseradish peroxidase-conjugated secondary antibodies (Promega). Washes were performed in PBS containing 0.02% Tween. The signal was detected as Chemi Doc XRS+ (Bio-Rad). Signal intensity of immunoreactive bands was measured using Adobe Photoshop. Full scan images of the western blots and gels used in the main figures are shown in Supplementary Fig. 8. Unless otherwise specified, the experiments of western blotting were repeated at least three times. In Figs 3c,e and 4b, and Supplementary Figs 2c–e, 4b,c, 5a and 6a were repeated at least two times.

### Immunoprecipitation

For preparing whole-cell lysates of HEK293T cells, cells were washed by PBS and lysed in ice-cold lysis buffer. The lysates were vortexed for 40 min at 4 °C, and insoluble material was removed after centrifugation for 10 min. For immunoprecipitation of FLAG-tagged Plk4 proteins, whole-cell lysates were incubated with FLAG antibody-conjugated M2 agarose (Sigma) for 2 h at 4 °C. Since the expression levels of FLAG-Plk4 proteins in the cells were very low, we monitored them by using the Flag immunoprecipitation instead of using the input materials. For HsSAS-6 immunoprecipitation, whole-cell lysates were incubated with protein G sepharose for 1 h at 4 °C for preclear, and then incubated for 2 h at 4 °C with protein G agarose that had been incubated with anti-HsSAS-6 antibodies. In both cases, the beads were washed at least four times with lysis buffer and resuspended in SDS sample buffer before loading onto a SDS–PAGE gel.

### *In vitro* kinase assay and MBP pull-down assay

For *in vitro* kinase assay, HEK293T cells were transfected with Plk4ΔPEST-FLAG WT or kinase-dead using Lipofectamine 2000 (Invitrogen). After 24 h, cells were harvested, treated with lysis buffer (20 mM Tris/HCl, pH 7.5, 150 mM NaCl, 0.5% Triton X-100, 1 mM DTT, 2 mM MgCl_2_ and 1/1,000 protease inhibitor cocktail (Nacalai Tesque)) and the lysates were immunoprecipitated with beads conjugated to FLAG antibodies. The beads were washed four times with lysis buffer supplemented with additional 500 mM NaCl and twice with kinase buffer (20 mM Tris HCl (pH 7.5), 150 mM NaCl and 1 mM DTT). The beads were then incubated with bacterially expressed recombinant proteins of STIL fragments thereof in 30 μl kinase buffer containing 10 mM MgCl_2_ and 30 μM ATP without or with 5 μ Ci [γ-^32^P] ATP. Kinase reactions were performed at 30 °C for 15–90 min and terminated by adding SDS sample buffer. Proteins were separated by SDS–PAGE, stained with SimplyBlue Safe (Invitrogen) and phosphorylation was visualized by autoradiography (Typhoon FLA 9000, GE Healthcare). After the kinase reaction, the resulting materials were subsequently processed for *in vitro* binding assay with MBP-HsSAS-6 proteins. *In vitro* kinase assays for Fig. 3a and Supplementary Fig. 3b were repeated three times.

For *in vitro* MBP pull-down assays in Fig. 2d,e and Supplementary Fig. 2c, after the kinase reaction, the supernatant and eluted fraction with FLAG peptides (Sigma) both of which contained phosphorylated STIL N3C proteins were collected. For other *in vitro* MBP pull-down assays, only the supernatant was collected. The resulting fractions were then incubated with MBP-HsSAS-6 full length purified from baculovirus/insect cell expression system and thereafter pulled down using amylose resin (New England Biolabs). Input and the protein complexes pulled down with the resins were analysed by western blotting using STIL, HA, FLAG or HsSAS-6 antibodies.

DNAs encoding fragments of human STIL were cloned in pGEX system vectors (GE Healthcare) encoding for glutathione S-transferase (GST) tags. The recombinant protein expression of the fragments was performed in *E. coli* strain BL21 gold (DE3) in LB medium. Protein expression was induced at 22 °C by addition of 0.3 mM isopropyl-β-D-thiogalactoside and allowed to proceed for 18 h. Cell pellets were lysed by lysozyme treatment and sonication, resuspended in lysis buffer containing 50 mM Tris HCl (pH 7.5), 150 mM NaCl, 2 mM MgCl_2_, 5 mM EDTA, 1 mM DTT, 1:500 protease inhibitor cocktail (Nacalai Tesque) and 0.5% Triton X-100. The lysates were incubated with Glutathion sepharose beads (GE Healthcare). The beads were then washed 10 times with lysis buffer supplemented with additional 500 mM NaCl. For preparing STIL fragments, proteins were eluted from the beads by removal of the GST tags by PreScission Protease (GE Healthcare) in a cleave buffer containing 20 mM Tris HCl (pH 7.5), 150 mM NaCl and1 mM DTT. For *in vitro* MBP pull-down assays in Figs 2g and 4b, and Supplementary Figs 2e and 6c,d, GST-fused STIL fragments were eluted by 10 mM glutathione in the cleave buffer.

Since it was not feasible to obtain soluble fraction of GST-fused HsSAS-6 full-length proteins from bacteria, we generated MBP-fused HsSAS-6 full-length proteins using Baculovirus Expression System with Gateway Technology (Invitrogen). In brief, DNA encoding HsSAS-6 full length was cloned into a modified pENTR-1A-5Myc-MBP vector. The expression clone was obtained from the entry clone and a pDEST vector through gateway cloning strategy. The recombinant bacmid was then obtained from DH10Bac *E. coli* cells transformed with the expression vector, and subsequently transfected to Sf9 insect cells with Cellfectin II reagent (Invitrogen). The titre of recombinant baculovirus was amplified by repeated infection to Sf9 cells. MBP-5Myc-HsSAS-6 full-length proteins were purified from 1 l (~2 × 10^6^ ml^–1^) culture of the Sf9 cells infected with sufficiently amplified baculovirus for 3 days. The procedure for protein purification was similarly done with amylose resin as described above for GST-fusion proteins.

### Mass spectrometry

For MS analysis, to identify Plk4-phosphorylated residues of STIL, the STIL proteins phosphorylated by Plk4ΔPEST-FLAG *in vitro* were digested into shorter peptides in solution by trypsin. The peptides were subsequently desalted and analysed by a nanoLC-linear ion trap-orbitrap mass spectrometer. MS analysis was repeated two times.

### Yeast two-hybrid analysis

Yeast strain L40 (a gift from Masato Kanemaki) was grown in complete medium (yeast extract peptone dextrose; (YPD)) and transformed with a modified version of the vectors pSM671 (bait) and pSM378 (prey; gifts from Satoru Mimura) that contained full length or fragments of Plk4 or STIL. Positive colonies were cultured on yeast plate without leucine and tryptophan (SD–L/–W) in the presence of histidine overnight. On the next day, cells were streaked on SD–L/–W without histidine plates supplemented with 50 mM 3-amino-triazol. Two independent colonies were streaked per sample. Plates were placed at 30 °C for 3 days. Yeast two-hybrid analysis was repeated at least three times.

## Author contributions

M.Oh. and D.K. designed the study; M.Oh., T.A., Y.N. and D.K. performed experiments; H.K.-H. and M.Oy. performed mass spectrometry analysis; H.G. and M.I. provided reagents for the baculovirus/sf9 expression system; M.Oh. and D.K. designed experiments and analysed data; and M.Oh. and D.K. wrote the manuscript, which was commented on by all authors.

## Additional information

**How to cite this article:** Ohta, M. *et al.* Direct interaction of Plk4 with STIL ensures formation of a single procentriole per parental centriole. *Nat. Commun.* 5:5267 doi: 10.1038/ncomms6267 (2014).

## Supplementary Material

Supplementary Information Supplementary Figures 1-8
