# Supplementary material for: Direct interaction of Plk4 with STIL ensures formation of a single procentriole per parental centriole
Source: Nat Commun. 2014 Oct 24;5:5267. doi: 10.1038/ncomms6267 (PMC4220463; doi:10.1038/ncomms6267)
Supplement: Supplementary Information — Supplementary Figures 1-8 [file ncomms6267-s1.pdf]

Supplementary Figure 1

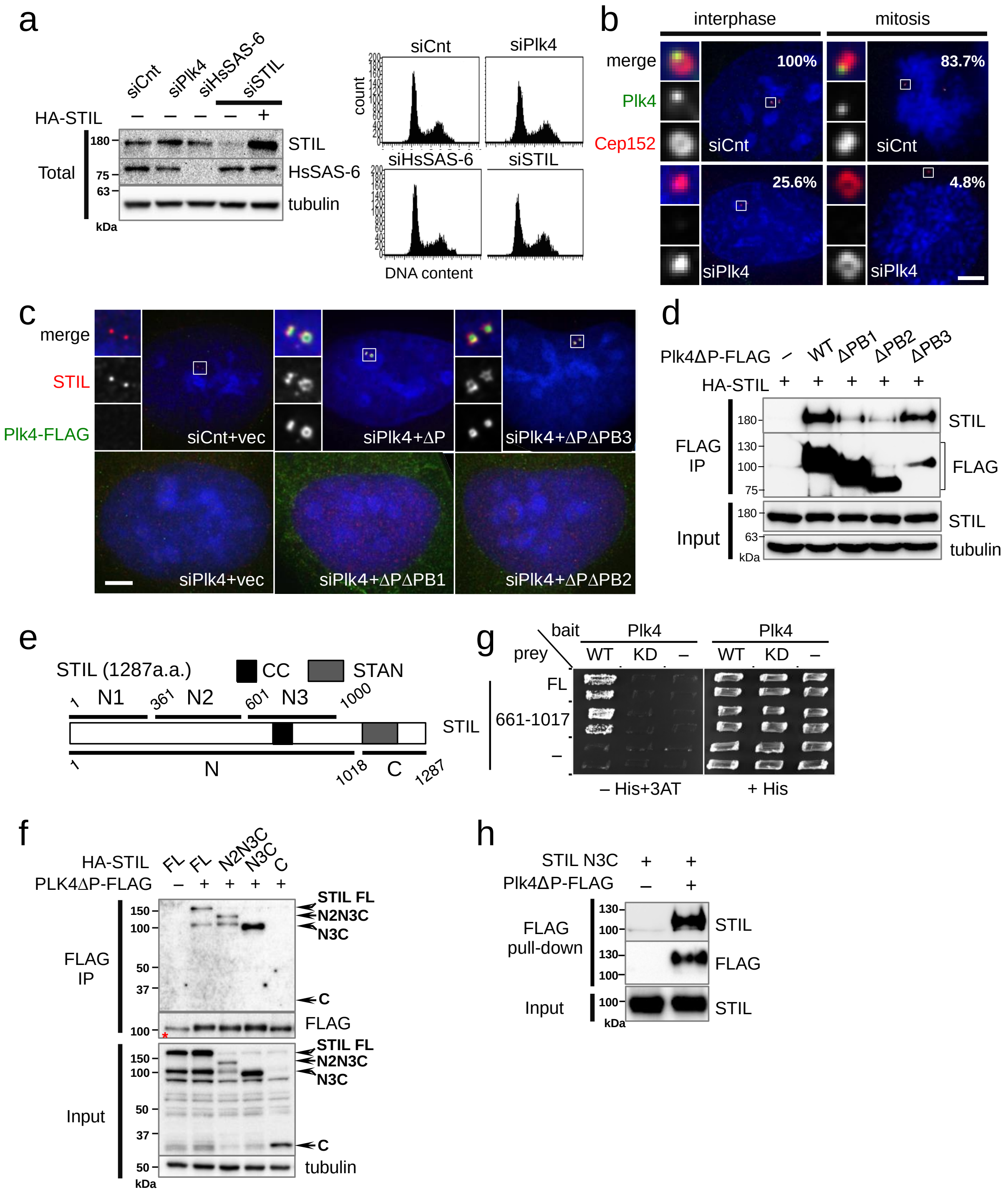

Supplementary Figure 1. Direct interaction between Plk4 and STIL. (a) U2OS cells or U2OS cells expressing HA-STIL were treated with control siRNAs or siRNAs targeting the 3'UTR of Plk4, STIL or HsSAS-6 for 48 hours. Total cell lysates were analyzed by western blotting using the indicated antibodies. The DNA content of cells was monitored by flow cytometry. **(b)** U2OS cells were treated with control siRNAs or siRNAs targeting the 3'UTR of Plk4. The cells were stained with antibodies against Plk4 (green) and Cep152 (red). DNA is shown in blue. The values are mean percentages of centrosomes with Plk4 from three independent experiments (N > 60 for interphase cells, N > 20 for mitotic cells). Insets show approximately nine-fold magnified views. Scale bar: 5 µm. **(c)** U2OS cells were treated with control siRNAs or siRNAs targeting 3'UTR of endogenous Plk4 for 24 hours, followed by transfection with an empty vector, Plk4ΔPEST-FLAG wild-type or deletion constructs (ΔPB1 (a.a. 586-699), ΔPB2 (a.a. 700-887) and ΔPB3 (a.a. 888-970)) for 24 hours. The cells were immunostained with antibodies against STIL and FLAG. Insets show approximately three-fold magnified views. Scale bar: 5 µm. Note that the two tandem polo boxes, PB1 and PB2, but not the C-terminal polo box (PB3), are needed for centriolar targeting of Plk4 and STIL. The experiment was repeated at least two times. **(d)** HEK 293T cells expressing Plk4ΔPEST-FLAG wild-type (WT), ΔPB1, ΔPB2 or ΔPB3 were immunoprecipitated with FLAG antibody. Total cell lysates and immunoprecipitates (IPs) were analyzed by western blotting using the indicated antibodies. Note that when either PB1 or PB2 was deleted, the binding of the Plk4 mutant proteins to endogenous STIL was compromised as compared with that of Plk4 WT or ΔPB3. Considering that the Plk4 PB1-PB2 domains that are essential for the formation of the Plk4 homodimer share a unique interdomain arrangement, it is possible that the full PB1-PB2 cassette is critical for the direct recognition of STIL. **(e)** Schematic of STIL full length (FL) and fragments. CC: coiled-coil, a.a. 721-746, STAN: a.a. 1061-1147. **(f)** HEK293T cells coexpressing Plk4ΔPEST-FLAG and HA-STIL FL, N2N3C (a.a. 361-1287), N3C (a.a. 601-1287) or C (a.a. 1018-1287) were immunoprecipitated with FLAG antibody. Total cell lysates and IPs were analyzed by western blotting using STIL, FLAG or tubulin antibodies. Asterisk: non-specific band. **(g)** Yeast two-hybrid assay was performed as in Figure 1e except for using the plate with histidine (+His) as the control. **(h)** *In vitro* binding assay testing direct interaction between Plk4ΔPEST-FLAG purified from HEK293T cells and bacterially-purified STIL N3C. STIL N3C proteins were incubated with or without Plk4ΔPEST-FLAG and subsequently pulled down with FLAG antibody. Total cell lysates and IPs were analyzed by western blotting using STIL or FLAG antibodies.

Supplementary Figure 2

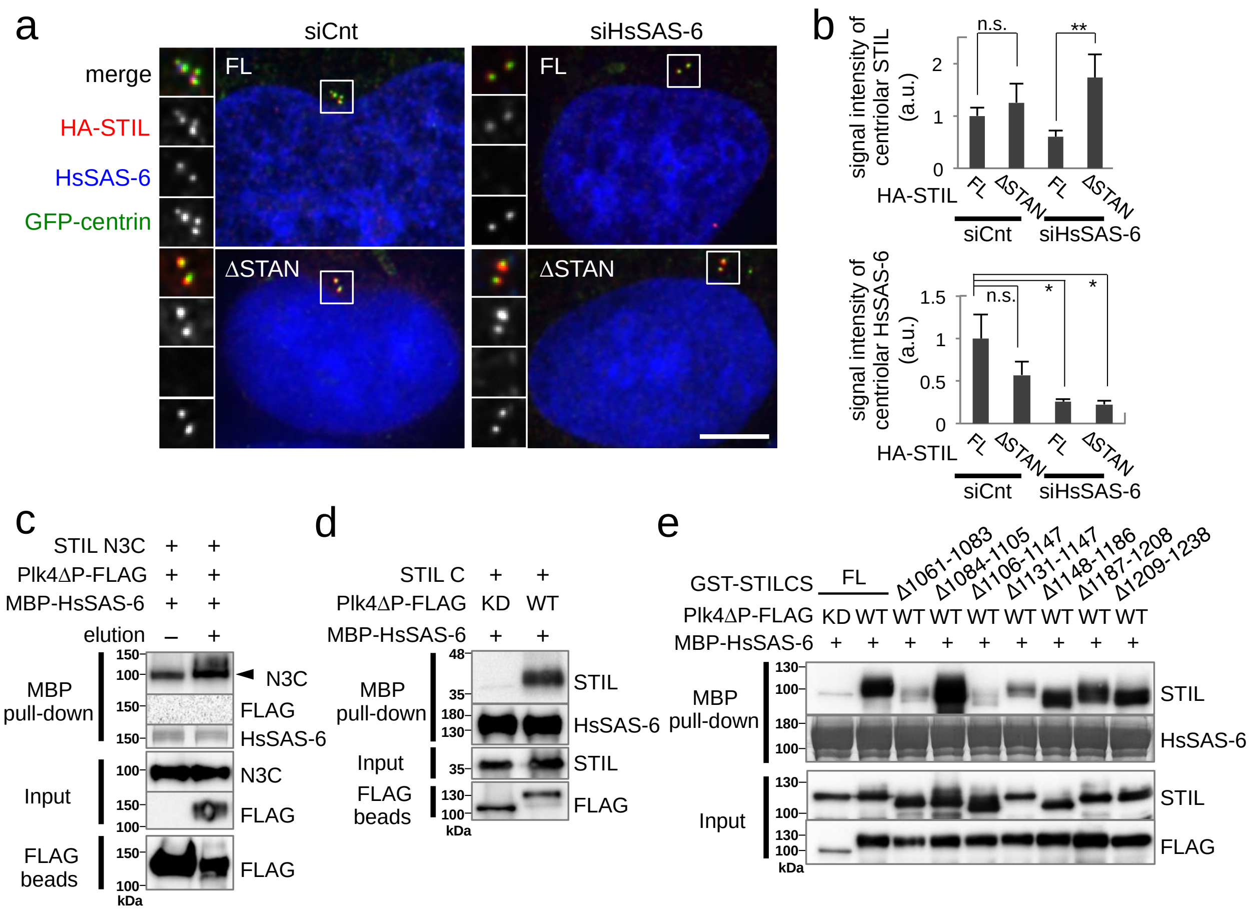

Supplementary Figure 2. STIL STAN motif is crucial for HsSAS-6 centriolar targeting and STIL/HsSAS-6 interaction. (a) U2OS cells stably expressing GFP-centrin1 were treated with control siRNA or siRNA targeting 3'UTR of endogenous HsSAS-6, followed by transfection with HA-STIL full length (FL) or ΔSTAN (Δ1061-1147 a.a.). The cells were stained with antibodies against HA, HsSAS-6 and GFP. DNA is shown in blue. Insets show approximately three-fold magnified views. Scale bar: 5 µm. The experiment was repeated at least two times. **(b)** Histograms represent the relative signal intensity of centriolar STIL (top) or HsSAS-6 (bottom) subtracted by that of the cytoplasmic signal. Values are mean percentages ± standard error of mean (SEM) (N = 10 for control siRNA-treated cells, N = 21 for HsSAS-6 siRNA-treated cells). *P < 0.05, **P < 0.01, n.s., not significant (one tailed t-test). Note that although it has been previously reported that endogenous STIL cannot localize to centrioles upon depletion of HsSAS-6, exogenous STIL FL proteins can localize to centrioles even in the

absence of HsSAS-6. These observations presumably stem from the higher expression levels of exogenous STIL proteins compared to those of the endogenous proteins. We found that STIL ΔSTAN tended to localize more stably at the centrioles as compared to STIL FL, even in the absence of endogenous HsSAS-6. Although the reasons for this are unknown, we speculate that HsSAS-6 is likely needed for the maintenance and/or stability of STIL at the centrioles rather than its centriolar recruitment. **(c)** Plk4-phosphorylated STIL N3C directly interacts with MBP-HsSAS-6 in the absence of Plk4ΔPEST-FLAG. *In vitro* binding assay was performed as described in Fig. 2c. After the kinase reaction, only the supernatant containing phosphorylated STIL N3C (a.a. 601-1287) was collected for the left lane, and the supernatant and eluted fraction with FLAG peptides were collected for the right lane. The resulting fractions were thereafter incubated with MBP-HsSAS-6 for *in vitro* binding assay. Input and the protein complexes pulled-down with amylose resin or FLAG-beads were analyzed by western blotting using STIL or FLAG antibodies or by Simply Blue Safestaining. Note that, in both conditions with and without FLAG-peptide elution, STIL that was phosphorylated by Plk4ΔPEST-FLAG directly bound to MBP-HsSAS-6. **(d)** *In vitro* kinase and binding assays were performed as in the left lane of (c) using STIL C fragment (a.a. 1018-1287). **(e)** *In vitro* kinase and binding assays were performed as described in (d) except for using recombinant GST-STIL CS (a.a. 1051-1287) FL or deletion mutant proteins. MBP-HsSAS-6 was detected by Simply Blue Safestaining.

Supplementary Figure 3

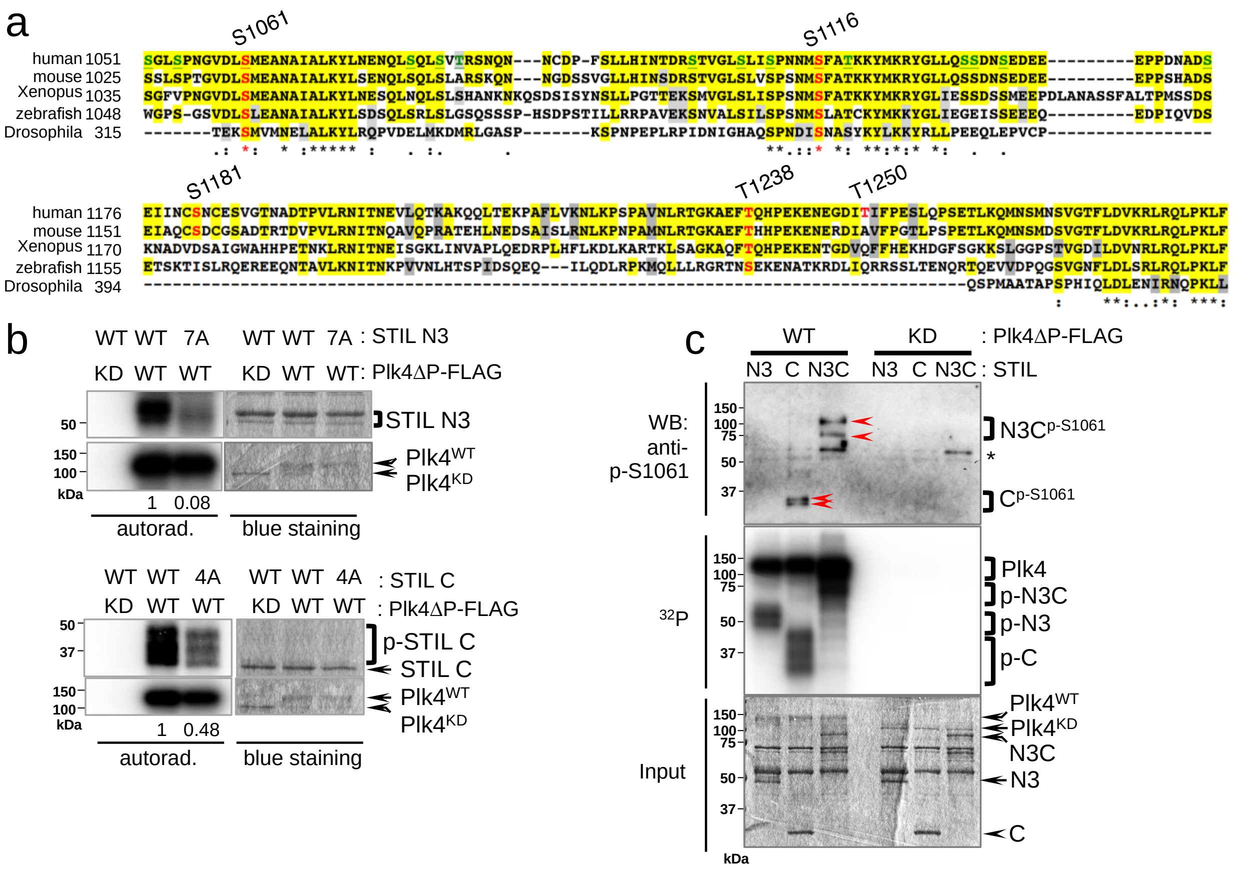

Supplementary Figure 3. Phosphorylation sites of STIL by Plk4 identified by mass spectrometry or phospho-specific antibodies. (a) Alignment of the C-terminal region of STIL within human, mouse, Xenopus and zebrafish STIL, and Drosophila Ana2. Colors and symbols indicate the same as in Figure 1c. The S/T sites tested in Figure 4b are shown in green. The positions of the phosphorylation sites identified by mass spectrometry or using phospho-specific antibodies are indicated in red. **(b)** Recombinant STIL N3 (a.a. 601-1000) wild-type (WT) and the 7A non-phosphorylatable mutant (top), STIL C (a.a. 1018-1287) WT and the 4A non-phosphorylatable mutant (mutated at S1116, S1181, T1238 and T1250 to alanine; bottom) proteins were bacterially-purified, and were incubated with Plk4ΔPEST-FLAG WT or kinase-dead (KD) proteins purified from HEK293T cells for *in vitro* kinase assay. The incorporation of [γ-^{32}P] ATP to the substrates was visualized by autoradiography, and the loaded proteins were monitored by Simply Blue Safestaining. The values

indicate the relative intensity of the phosphorylated STIL bands. Note that mutation of the seven serine/ threonine residues in STIL N3 or the four serine/ threonine residues in STIL C to alanine significantly dampened phosphorylation by Plk4ΔPEST-FLAG. **(c)** Plk4 phosphorylates STIL at Serine 1061 *in vitro*. Recombinant STIL N3C (a.a. 601-1287), STIL N3, STIL C proteins were incubated with Plk4ΔPEST-FLAG WT or KD proteins purified from HEK293T cells for *in vitro* kinase assay. The total reaction mixture was thereafter analyzed by SDS-PAGE, followed by Simply Blue Safestaining and autoradiography or western blotting using antibodies against phospho-S1061. Red arrowheads indicate the phosphorylated STIL fragments at S1061. Asterisk: a non-specific band.

Supplementary Figure 4

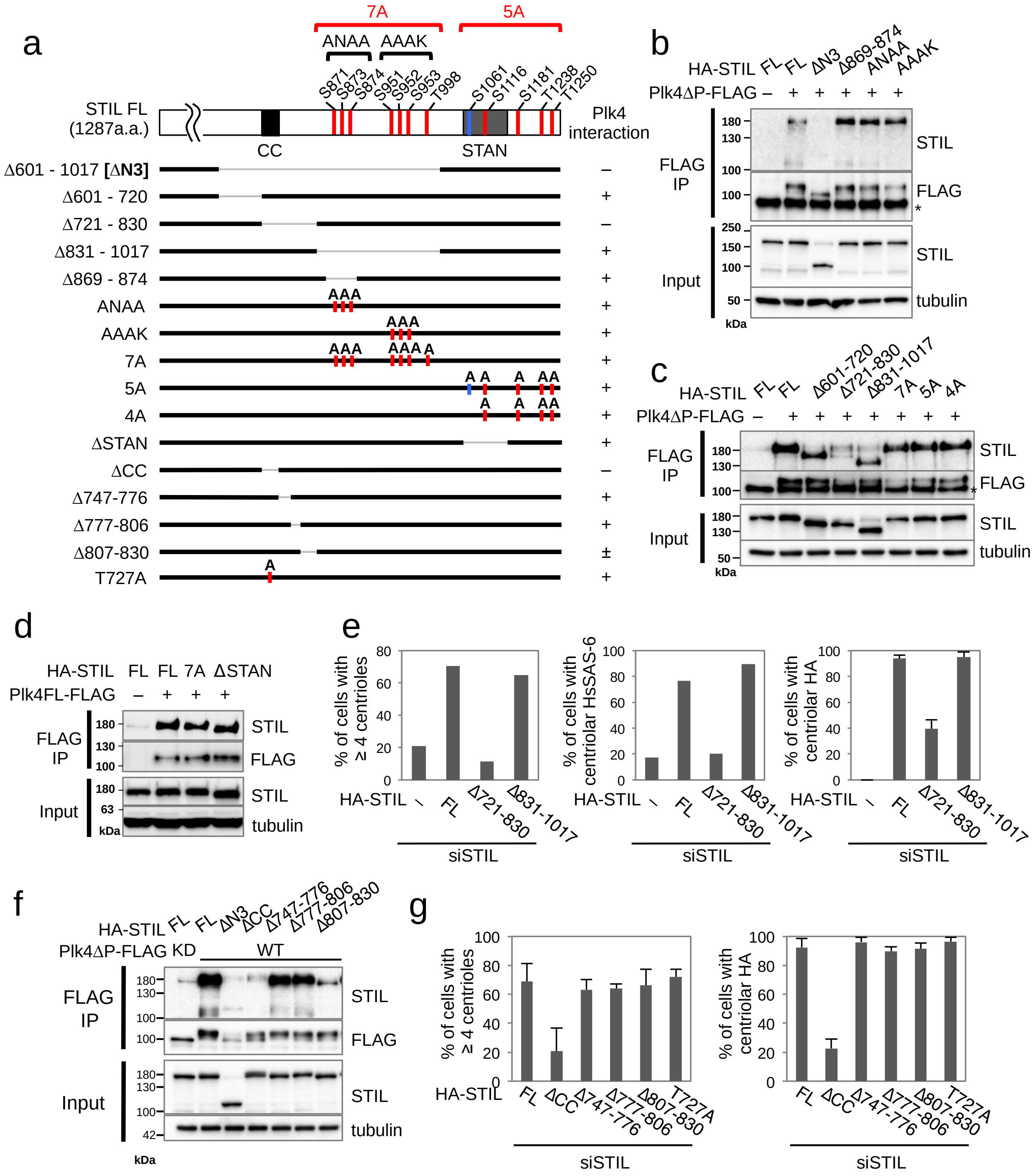

Supplementary Figure 4. Phosphorylation of STIL by Plk4 is most likely dispensable for the Plk4/STIL interaction. (a) Schematic of STIL full length (FL) and the summary of the interactions between the indicated STIL mutant proteins and Plk4ΔPEST examined in this figure. Plk4-phosphorylated serine/threonine residues of STIL identified by mass spectrometry or phospho-specific antibodies and the alanine substitution mutants ANAA, AAAK, 7A, 5A and 4A are shown. T727 is the sole S/T residue within the CC domain. **(b,c)** Mutating the Plk4-phosphorylated residues in STIL N3 or STIL C did not affect the interaction between Plk4 and STIL. HEK293T cells co-expressing Plk4ΔPEST-FLAG and the indicated HA-STIL FL, deletion mutants or non-phosphorylatable mutant proteins indicated in (a) were immunoprecipitated with FLAG antibody. Total cell lysates and immunoprecipitants (IPs) were analyzed by Western blotting using the indicated antibodies. Asterisk: a non-specific band. **(d)** Co-IP assays testing interactions between the indicated HA-STIL proteins and Plk4 FL. HEK293T cells expressing the empty FLAG vector (−) or FLAG-tagged Plk4 FL and HA-STIL FL, 7A or ΔSTAN were immunoprecipitated with FLAG antibodies. Total cell lysates and IPs were analyzed by western blotting using the indicated antibodies. **(e)** U2OS cells were treated with siRNAs targeting 3'UTR of endogenous STIL, followed by transfection with an empty vector, HA-STIL FL, Δ721-830 or Δ831-1017 vector. The cells were immunostained with antibodies against centrin, HsSAS-6 and HA. Histograms represent mean percentages from two independent experiments for centriolar centrin and HsSAS-6 counting, and mean percentages ± SD from four independent experiments for centriolar HA counting (N > 50). **(f)** HEK293T cells expressing Plk4ΔPEST-FLAG wild-type or kinase dead and the indicated HA-STIL FL or deletion mutants were immunoprecipitated with FLAG antibodies. Total cell lysates and IPs were analyzed by western blotting using the indicated antibodies. The left three lanes are also shown in Figure 6e. **(g)** U2OS cells were treated with STIL 3'UTR siRNAs, followed by transfection with the vectors encoding the indicated HA-STIL FL or deletion mutants or non-phosphorylatable mutant (T727A). The cells were immunostained with antibodies against centrin and HA. Histograms represent mean percentages ± SD of interphase cells in each condition from three independent experiments (N > 50).

Supplementary Figure 5

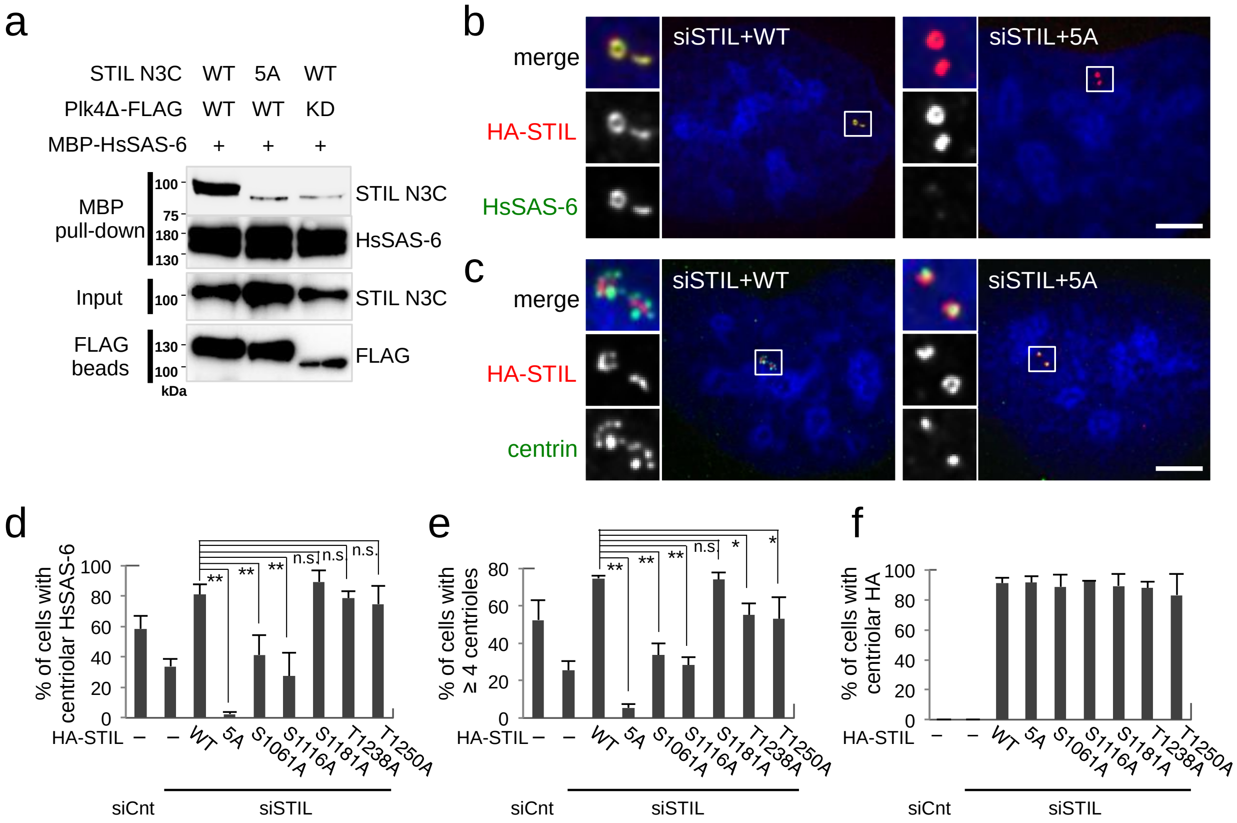

Supplementary Figure 5. Functional analysis of phosphorylation sites within the STIL C-terminal region identified by mass spectrometry. (a) *In vitro* kinase and binding assays were performed as described in Figure 2c, using STIL N3C wild-type (WT) or non-phosphorylatable 5A mutant fragment. Note that the phosphorylation of the C-terminal region of STIL was crucial for direct binding to MBP-HsSAS-6. **(b-f)** U2OS cells were treated with control siRNA or siRNA targeting the 3'UTR of endogenous STIL, followed by transfection with an empty vector (–), HA-STIL WT or non-phosphorylatable mutants, 5A (mutated at S1061, S1116, S1181, T1238 and T1250 to alanine), S1061A, S1116A, S1181A, T1238A and T1250A. The cells were fixed and immunostained with antibodies against HA and HsSAS-6 (b) or centrin (c). DNA is shown in blue. Histograms represent frequency of interphase cells with centriolar HsSAS-6 (d) or with ≥ 4 centrioles (e) or with centriolar HA (f) in each condition. Insets show approximately seven-fold magnified views. Scale bar: 5 μm. Values are mean percentages ± SD from three independent experiments (N > 50 for each condition). *P < 0.05, **P < 0.01, n.s., not significant (one tailed t-test).

Supplementary Figure 6

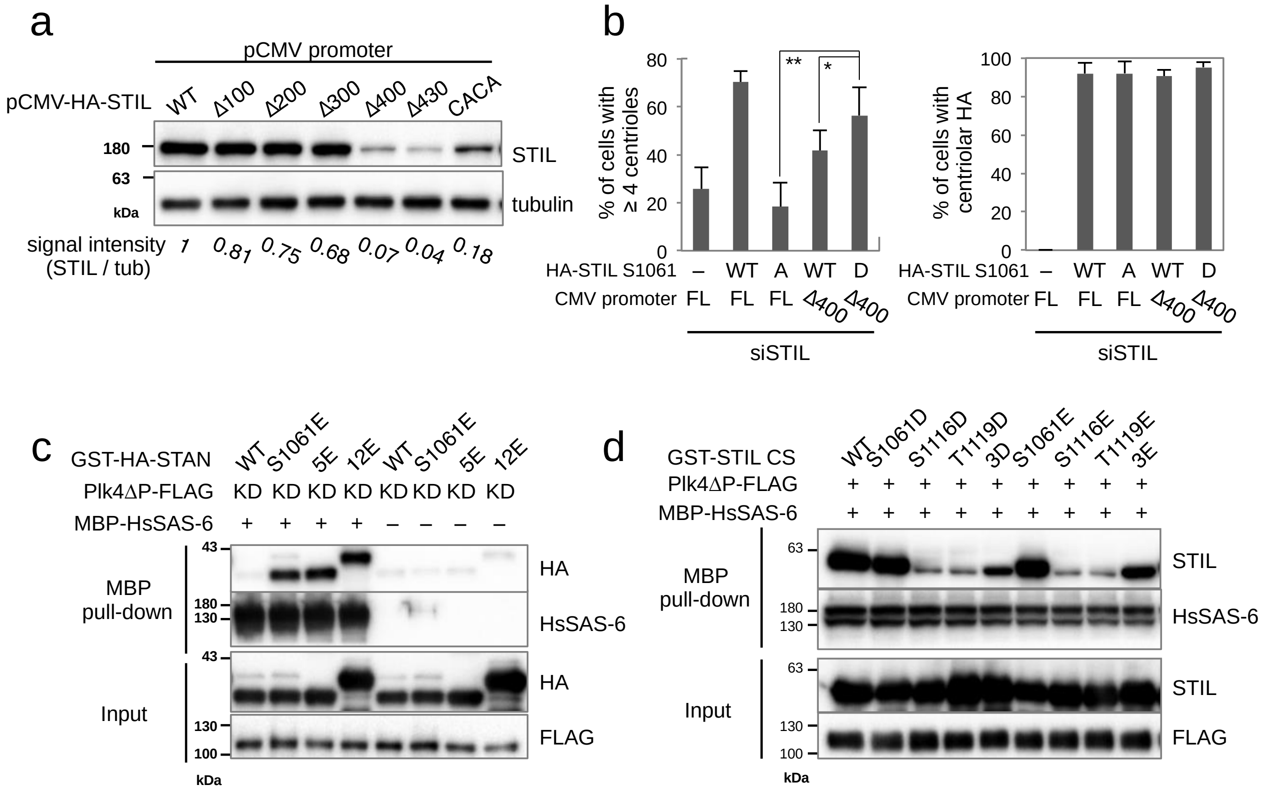

Supplementary Figure 6. Characterization of the phosphorylation sites within the C-terminal region of STIL for the STIL/HsSAS-6 interaction and procentriole formation. (a) Optimization of exogenous STIL expression levels under the modified CMV promoter in U2OS cells. The conventional CMV promoter was modified by deleting 100-430 base pairs from the 5' end, or replacing the TATA box with the CACA box. **(b)** U2OS cells were treated with control siRNA or siRNA targeting the 3'UTR of endogenous STIL, followed by transfection with an empty vector (–), HA-STIL wild-type (WT), non-phosphorylatable (S1061A) or phosphomimetic (S1061D) mutant. The expression of HA-STIL WT and S1061D was induced under an attenuated CMV promoter by which expression levels of STIL were decreased by several times. The cells were immunostained with antibodies against HA and centrin. Histograms represent frequency of interphase cells with ≥ 4 centrioles or with centriolar HA in each condition. Values are mean percentages ± SD from four independent experiments (N > 50 for each condition). *P < 0.05, **P < 0.01, n.s., not significant (one tailed t-test). **(c)** *In vitro* binding assays to test the interaction between the STIL STAN WT or phosphomimetic mutant proteins and HsSAS-6. Bacterially-purified GST-HA-STIL

STAN WT, S1061E, 5E (S1061 and the 4 S residues found to be phosphorylated *in vivo* (S1108, S1111, S1132 and S1135, reported in PhosphoSitePlus)) or 12E (all the S/T residues within the critical parts of the STAN motif (see in Figure 2f and 4a) except for S1116 and T1119: namely, S1051, S1054, S1061, S1078, S1081, T1083, S1108, S1111, S1131, S1132, S1135, S1147) recombinant proteins were pre-incubated with Plk4ΔPEST-FLAG kinase-dead (KD) proteins. The resulting supernatant was then incubated with MBP-HsSAS-6 as described in Figure 2C. Input and the pulled-downed complexes were analyzed by western blotting using indicated antibodies. **(d)** *In vitro* kinase and binding assays were performed as described in (c) except for using the indicated GST-STIL CS (a.a. 1051-1287) WT or phosphomimetic mutant fragment. S1061, S1116 and T1119 were identified as critical residues for the STIL/HsSAS-6 interaction by the alanine mutational scan described in Figure 4b. All of these sites are mutated to aspartic acid or glutamic acid in 3D/E mutants. Note that mutating S1116 or T1119 to D/E disrupted the Plk4-mediated STIL/HsSAS-6 interaction *in vitro*.

Supplementary Figure 7

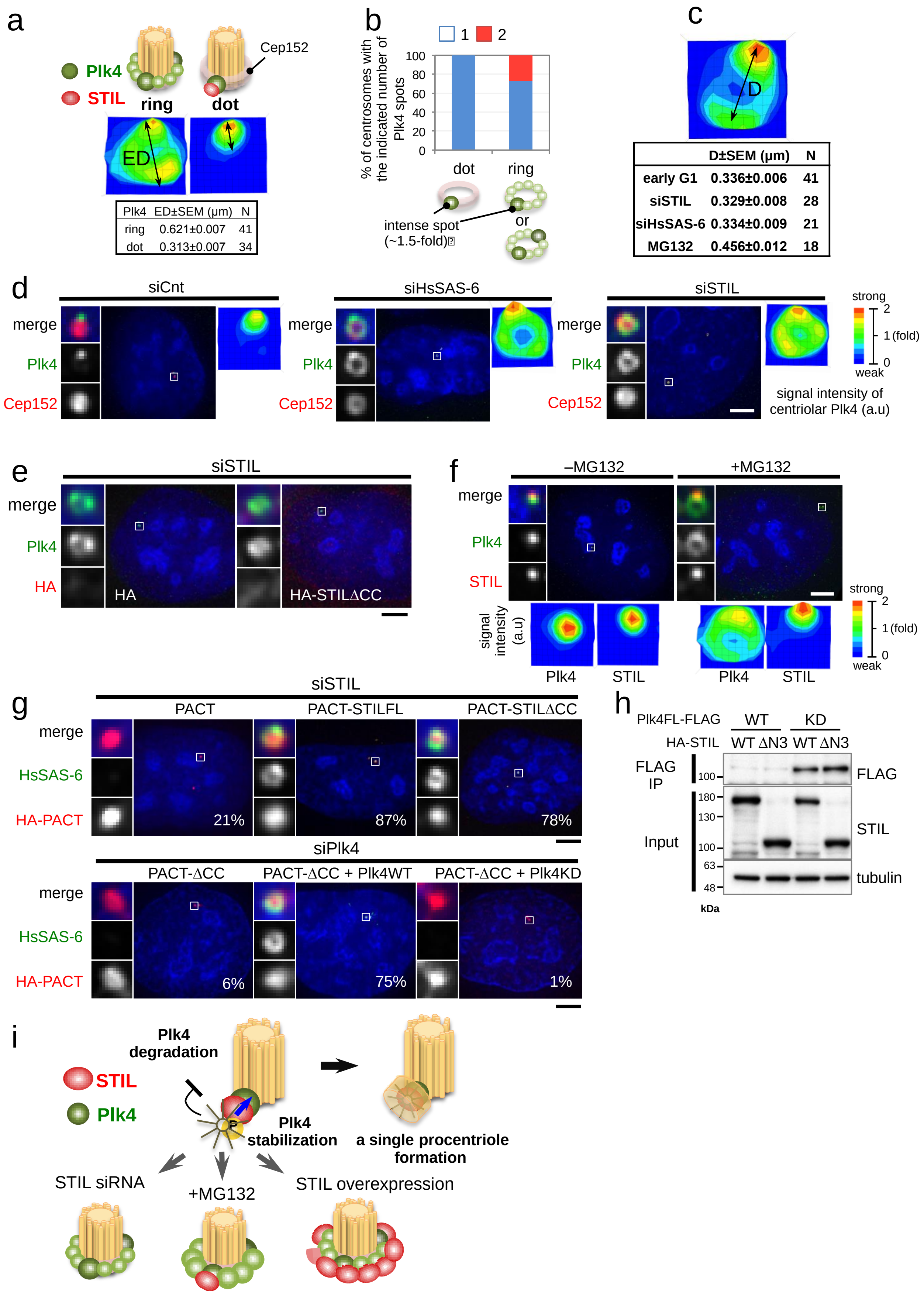

Supplementary Figure 7. Plk4 localizes to the centriole in a bimodal manner.
(a) The external diameter of centriolar Plk4 rings or dots was measured. Values are mean percentages ± SEM. ED: external diameter, N: number of total centrosomes. **(b)** The number of centrosomes harboring the indicated number of centriolar Plk4 spots with signal intensity above the threshold of 1.5-fold with respective to the average intensity was counted. N = 34 for Plk4 dots, N = 44 for Plk4 rings from three independent experiments. We also noted that the ring-like pattern of centriolar Plk4 mostly contains one (~72%) or two (~28 %) spots with relatively high signal intensity. It is therefore possible that such Plk4 spots give favorable sites for centriolar loading of STIL. **(c)** Distance between the polar central points of a Plk4 ring is measured as the diameter [D]. N: number of total centrosomes analyzed. Note that the diameter of a ring in the cells depleted of STIL or HsSAS-6 is comparable with that in the cells in early G1 phase, but less than that in the cells treated with MG132, reflecting the fact that centriolar Plk4 rings appeared to be more complete with MG132 treatment. **(d)** More examples of Figure 5d. Scale bars in the figure indicate 5μm. **(e)** U2OS cells treated with siRNAs targeting STIL-3'UTR and expressing HA empty-vector (HA), HA-STILΔCC (Δ721-746 a.a.) were stained with antibodies against Plk4 and HA. **(f)** More examples of Figure 6a. **(g)** U2OS cells treated with siRNAs targeting STIL-3'UTR and expressing HA-PACT, HA-PACT-STIL full-length (FL) or HA-PACT-STILΔCC (upper panels) or U2OS cells treated with siRNAs targeting Plk4-3'UTR and expressing Plk4 FL-FLAG wild-type (WT) or the kinase-dead (KD) together with HA-PACT-STILΔCC (lower panels) were stained with antibodies against HsSAS-6 and HA. The values indicate percentages of centrosomes with centriolar HsSAS-6 (N>60). Note that HA-PACT-STILΔCC recruits HsSAS-6 to centrioles, depending on Plk4 kinase activity. **(h)** HEK293T cells co-expressing Plk4 FL-FLAG WT or KD and HA-STIL FL or STIL ΔN3 were immunoprecipitated with FLAG antibodies. Total cell lysates and IPs were analyzed by western blotting using indicated antibodies. Note that the expression levels of Plk4 FL were strictly restricted through protein degradation mediated by trans-autophosphorylation. Therefore, there was a very small amount of activated Plk4 FL whereas the expression levels of the kinase-dead were much higher. Since STIL preferentially binds to and protects activated Plk4, unactivated Plk4 FL-WT proteins, a majority of Plk4 FL-WT expressed in the cells, were not stabilized by co-expression of HA-STIL FL. **(i)** The bimodal distribution of Plk4 at the centrioles was regulated by its interaction with STIL and protein degradation. Model suggesting how centriolar Plk4 is stabilized as a ring in the cells treated with STIL siRNA, proteasome inhibitor (MG132) or overexpressing STIL.

Supplementary Figure 8

Figure 1a

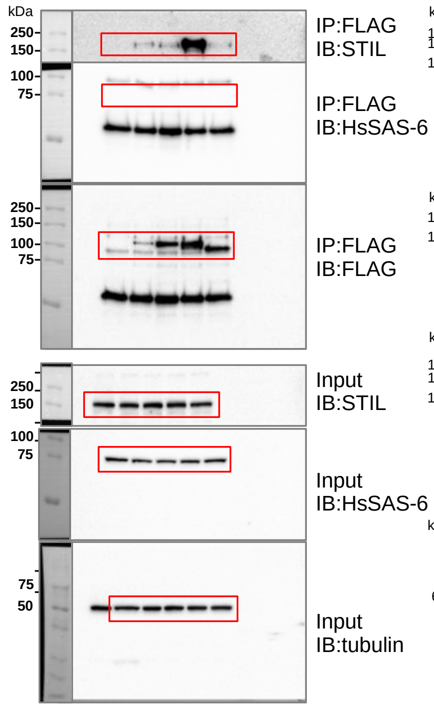

Figure 1d

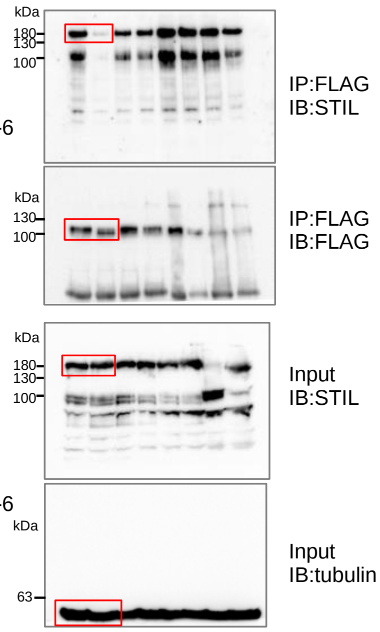

Figure 2b

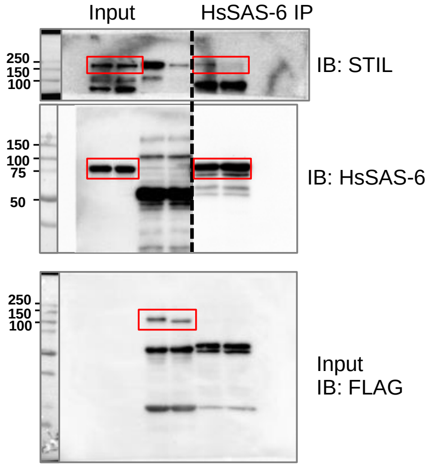

Figure 2d

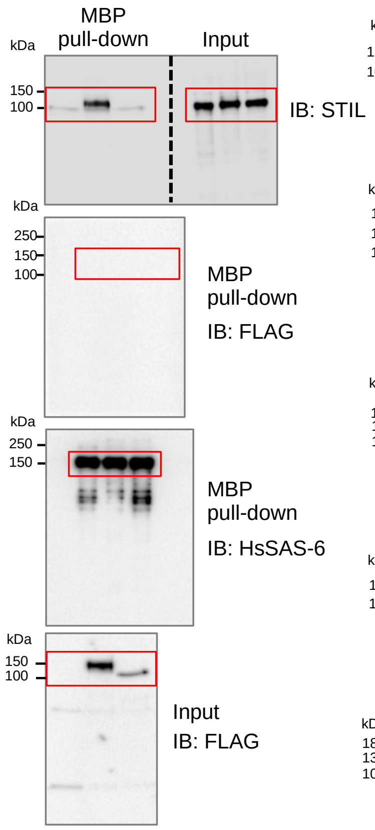

Figure 2e

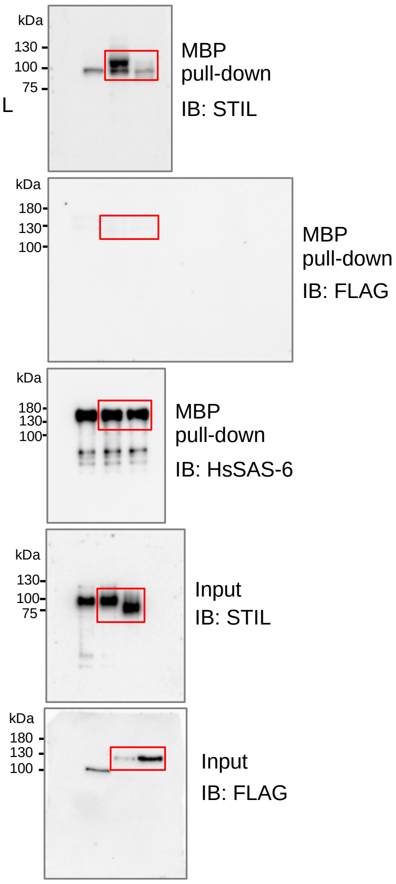

Figure 2g

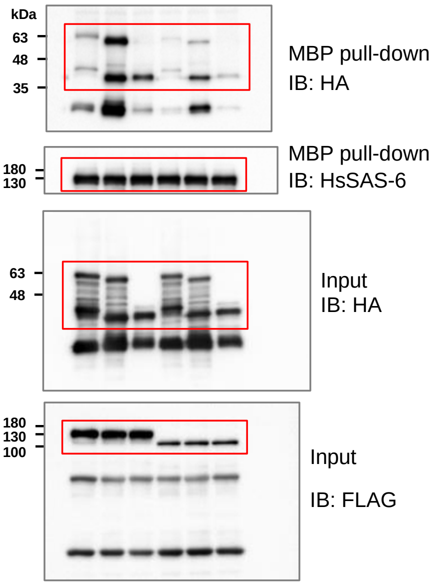

Supplementary Figure 8 continued

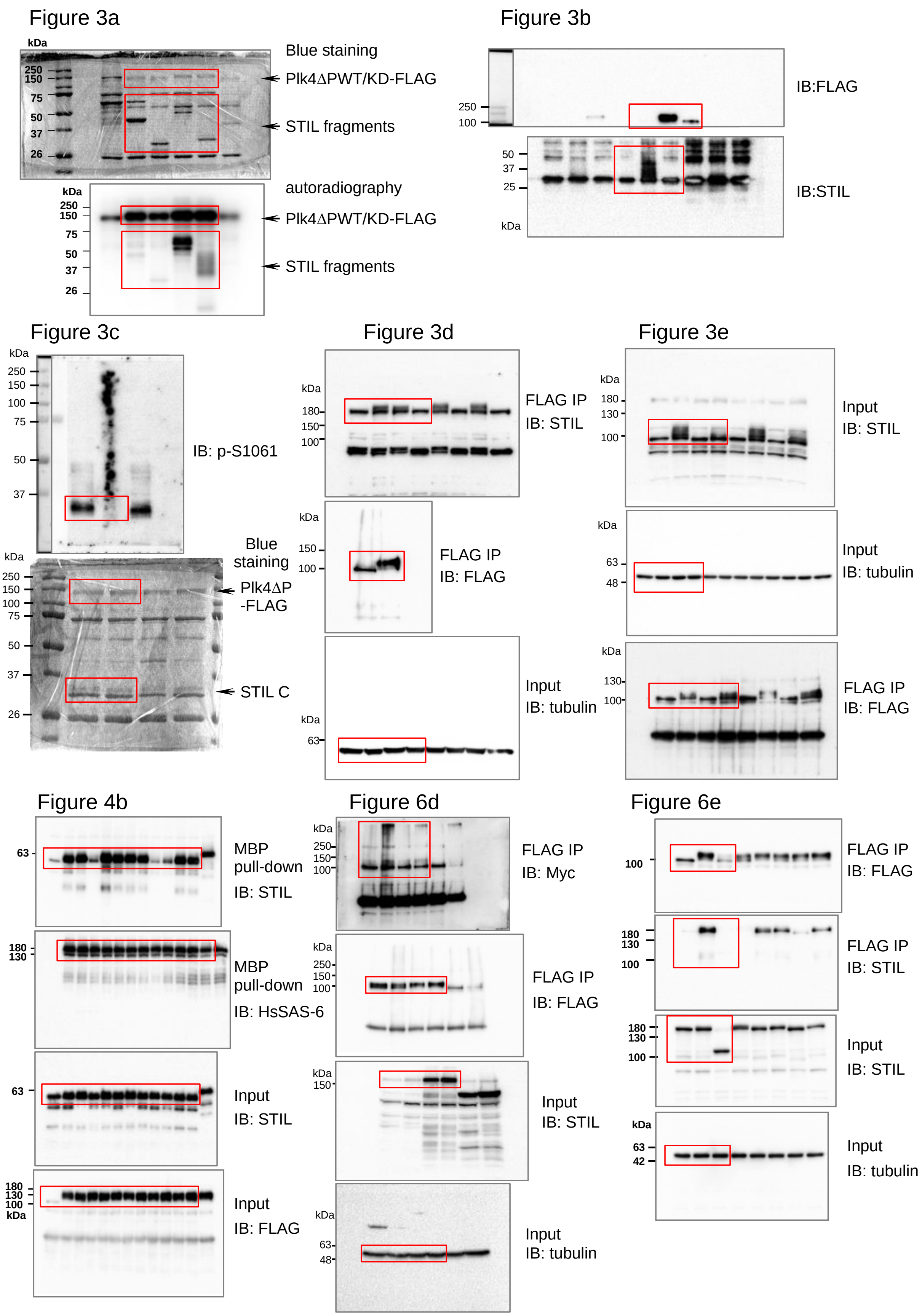

Supplementary Figure 8. Uncropped images of blots presented in the main paper. Red boxes indicate the cropped regions. Molecular weight markers are indicated in kDa. IB; immunoblot, IP; immunoprecipitate
